# Machine learning to predict morphology, topography and mechanical properties of sustainable gelatin-based electrospun scaffolds

**DOI:** 10.1038/s41598-024-71824-2

**Published:** 2024-09-09

**Authors:** Elisa Roldán, Neil D. Reeves, Glen Cooper, Kirstie Andrews

**Affiliations:** 1 Department of Engineering, Faculty of Science & Engineering, Manchester Metropolitan University, Manchester, M1 5GD UK; 2 Lancaster Medical School, Faculty of Health and Medicine, Lancaster University, Lancaster, LA1 4YW UK; 3 School of Engineering, University of Manchester, Manchester, M13 9PL UK

**Keywords:** Biomaterials, Nanoscale materials, Biomedical engineering, Mechanical engineering

## Abstract

Electrospinning is an outstanding manufacturing technique for producing nano-micro-scaled fibrous scaffolds comparable to biological tissues. However, the solvents used are normally hazardous for the health and the environment, which compromises the sustainability of the process and the industrial scaling. This novel study compares different machine learning models to predict how green solvents affect the morphology, topography and mechanical properties of gelatin-based scaffolds. Gelatin-based scaffolds were produced with different concentrations of distillate water (dH_2_O), acetic acid (HAc) and dimethyl sulfoxide (DMSO). 2214 observations, 12 machine learning approaches, including Generalised Linear Models, Generalised Additive Models, Generalised Additive Models for Location, Scale and Shape (GAMLSS), Decision Trees, Random Forest, Support Vector Machine and Artificial Neural Network, and a total of 72 models were developed to predict diameter of the fibres, inter-fibre separation, roughness, ultimate tensile strength, Young’s modulus and strain at break. The best GAMLSS models improved the performance of R^2^ with respect to the popular regression models by 6.868%, and the MAPE was improved by 21.16%. HAc highly influenced the morphology and topography; however, the importance of DMSO was higher in the mechanical properties. The addition of the morphological properties as covariates in the topographic and mechanical models enhanced their understanding.

## Introduction

Electrospinning is characterised for being a powerful manufacturing technique to create nano-micro-scaled fibre structures. This technique exhibits multiple advantages such as high surface area to volume ratio, tailored structures, ease of fibre functionalisation, possibility of use of a large variety of polymers and combinations, relatively low cost, and easy process. However, it often requires non-eco-friendly solvents that might be toxic, flammable, or difficult to dispose of and recycle, compromising the process's sustainability^1,2^.

One of the most popular natural polymers used in biomedical, pharmaceutical and food packaging applications is gelatin^3^. This polymer is synthesised from the hydrolysis of collagen, which is the most plentiful protein in the extracellular matrix. Due to that, gelatin is bioactive and contains arginine, glycine and aspartate integrin-binding motifs, which enhance cell adhesion and proliferation^4^. Moreover, its low cost, biodegradability, high biocompatibility and hydrophilicity make it attractive for tissue-engineered applications such as wound healing applications^5,6^, nervous system tissue^7–9^, dental applications^10^, bone tissue^11–14^ and skin tissue^15,16^, tendon implants^17^ and vascular grafts^18^. Despite all these advantages, gelatin solutions have the inconvenience of becoming gel at temperatures below 30 °C, which hinder the Taylor cone and fibre formation during electrospinning^19,20^. To overcome this problem, different solvents have been proposed, being the most common: Fluorinated alcohols such as 2,2,2-trifluoroethanol (TFE)^20,21^ or 1,1,1,3,3,3-hexafluoro-2-propanol (HIPF)^22^; dilutions of phosphate buffer saline (PBS) and ethanol^23,24^; carboxylic acids such as formic acid or acetic acid (HAc)^24–30^; mixtures of different solvents such as HAc and TFE^31^, HAc and dimethyl sulfoxide (DMSO)^31^, HAc and ethylene glycol^31^, HAc and formamide^31^ or HAc and ethyl acetate^27^.

Pharmaceuticals such as Pfizer^32^, GlaxoSmithKline^33,34^ or Sanofi^35^ have developed “traffic light” coded lists of solvents to assess the environmental, health, and safety (EHS) impact of solvents. According to those lists and the EHS indicator and Slater and Savelsky method^36^, the most eco-friendly solvents are water, alcohols, HAc, ketones, esters, or DMSO, acetonitrile and dimethyl propylene urea from the aprotic polar solvents group^2^.

Recent studies have demonstrated that the concentration of the polymer is the most important factor to predict morphological properties such as the diameter of the fibre^37–39^. In this study, our aim is to predict the influence of three green solvents’ concentration in the morphology (diameter of the fibres and inter-fibre separation), topography (roughness) and mechanical behaviour (ultimate tensile strength, Young’s modulus and strain at break) of gelatin-based electrospun scaffolds. After a preliminary study with HAc, PBS, denatured alcohol, ethanol, fetal bovine serum (FBS), DMSO and distilled water (dH_2_O), we determined that the solvents that produced scaffolds free of defects, homogeneous and with high-quality fibres were HAc, dH_2_O and DMSO^40^. In the present study, we investigate, through 12 machine learning models, these three green solvents using the *“ceteris paribus”* method^41^ to reduce the effect of interactions between input variables.

General linear models such as classic linear simple or multiple regressions, Multivariate Analysis of Variance (MANOVA) models or Multivariate analysis of Covariance (MANCOVA) models are popular prediction models in scientific publications. Many of these predictive statistical models used in electrospinning assumed that the dependent variable follows a normal probability distribution, without verifying it with relevant test (Kolmogorov–Smirnov or Shapiro–Wilk depending on the sample size)^38,42,43^. However, it was proved that not always variables such as diameter of the fibres or inter-fibre separation follow that distribution^39,44–46^ and therefore, an appropriate statistical analysis to find the most accurate prediction model should be done.

The Generalised Linear Model (GLM) generalises the linear regression allowing response variables with distributions belonging to the exponential family such as normal, binomial, Poisson, gamma or logistic^47^. Although these models were first time popularised by McCullagh and Nelder^48^ and are highly used in academia, they were never used to predict morphological, topographical and mechanical properties of electrospun scaffolds. The Generalised Additive Model (GAM) was developed as an extension of the GLM by Hastie and Tibshirani in 1986^49^. The GAM introduces non-linear smooth effects of the covariate on the dependent variable. In 2005, Rigby and Stasinopoulos^50^ proposed the Generalized Additive Models for Location, Scale and Shape (GAMLSS). These models assume that the response variable follows a distribution that can be not exponential, and the parameters of location, scale and shape can be flexibly and independently modelled. Although these models are very popular for their flexibility, versatility and interpretability, GAMs and GAMLSS have not been used to predict the morphology, topography and mechanical properties of electrospun scaffolds so far. Decision tree models (DT) are popular non-parametric supervised learning algorithms use in data mining^39^. Currently, just few recent studies investigated the used of this classification method for the electrospinning technique^37,39,51,52^; however, the effect of the solvent’s concentration on the morphology, topography and mechanical properties of electrospun scaffolds was not studied. Random forest (RF) is an extension of decision trees where the output of multiple decision trees is combined to predict a variable. Some work has been done using random forest to understand the mechanical stability in cellulose nanofibres^52^, to study the size particles in electrospraying^53^, predict size and bead formation in poly(vinylidene fluoride) nanofibres^54^ or predict the mechanical properties of 2D and 3D biomimetic electrospun scaffolds^51^; however, a study where six output variables are predicted with random forest was not performed yet. Support Vector Machine (SVM) is a supervised machine learning method used for classification, regression and outliers detection^55^. It is versatile, memory efficient and effective when the number of dimensions is higher than the number of samples^55^. The objective of this method is to find the hyperplane where the distance between data of different classes is maximised. By maximising the margin distance, test data can be more accurately classified. Recently, Kalantary et al^56^. used this method to predict the diameter of PCL/gelatin nanofibres, Trupp et al^54^. predicted the size and number of beads in poly(vinylidene fluoride) electrospun scaffolds, Pervez et al^57^. used it to predict the diameter of chitosan/polyvinyl alcohol nanofibers, Muqeet et al^52^. used it to predict the tensile strength of enhanced cellulose nanofibers and Roldan et al^51^. predicted the mechanical properties of electrospun scaffolds for tissue engineered application. In our study, SVM was used not only to predict the diameter of the fibres but also the inter-fibre separation, roughness, Young’s modulus, ultimate tensile strength and strain at break. Artificial Neural Network (ANN) is a non-linear predictive technique used in Big Data. Its popularity to predict fibre diameters in electrospun scaffolds has been recently increased^38,39,56,58,59^. However, its use to predict topographical and mechanical properties in those structures is still not extended.

This study provides a systematic (from less complex and flexible models to more complex and flexible ones) and novel methodology to find the most optimum models to predict the morphological (diameter of the fibres and inter-fibre separation), topographical (roughness), and mechanical properties (Young’s modulus, ultimate tensile strength and strain at break) of gelatin-based scaffolds manufactured with different concentrations of three green solvents. GLM, GAM, GAMLSS, decision trees, random forest, SVM and ANN are assessed and discussed for each dependent variable. This methodology can be adopted to predict endogenous variables for a wide range of applications.

## Materials and methods

### Materials

Gelatin powder type B from bovine skin (Bloom ~ 225 g) was purchased from Sigma Aldrich (UK). Glacial acetic acid (Sigma Aldrich, UK), DMSO (Sigma Aldrich, UK) and distilled water (dH_2_O) were used as solvents.

### Scaffold production

Nine solutions were prepared with 25% w/v of gelatin dissolved in concentrations of HAc and dH_2_O of 3:1, 1:1 and 1:3, adding 0%, 5% and 10% of DMSO. The scaffolds were fabricated with an electrospinning device (TL-01, NaBond, China) under the same set-up of manufacturing parameters following the *“ceteris paribus”* method^41^ to minimise the effect of interactions between input variables, which were fixed to 2 ml/h flow rate, 15 G needle, 26 kV, 15 cm diameter-rotating collector working at 1300 rpm, 11 cm distance between the needle and the collector, room temperature of 25 °C and 3 h spins time.

### Scaffold characterisation

#### Morphology of the fibres

The samples were visualised with a field emission scanning electron microscope Zeiss Supra 40 (FE-SEM, Carl Zeiss SMT Ltd., Cambridge, UK) following the process described in a previous study^60^. AxioVision SE64 Rel. 4.9.1 (Carl Zeiss SMT Ltd., Cambridge, UK) was used to obtain a total of 738 observations of diameter of the fibres and inter-fibre separation from the samples.

#### Topography of the scaffold

A total of 369 white light interferometry images were taken with an interferometer from ZeGage (Zygo Corporation, US), to measure the average roughness of the scaffolds as performed in a previous study^61^.

#### Mechanical characterisation

Quasi-static uniaxial tensile tests were performed until failure with a tensometer (Instron H10KS, US), 100 N load cell and 1 mm/min test speed to determine the mechanical properties such as Young’s modulus, ultimate tensile strength and strain at break for the samples produced with 25% w/v of gelatin dissolved in concentrations of HAc and dH_2_O of 3:1, 1:1 and 1:3, adding 0%, 5% and 10% of DMSO. A total of 1107 observations (369 observations/output variable) were obtained. A full description of the process has been documented in a recent study^61^.

### Prediction models

A systematic and novel methodology from less complex/flexible models to more complex/flexible models was used to predict the influence of green solvents in the morphological, topographical and mechanical properties of gelatin-based scaffolds produced with HAc/dH_2_O and DMSO. A total of 72 regression models were performed using GLM, GAM, GAMLSS, DT, RF, SVM and ANN to allow comparison between models.

General linear regression models were included in the study just to compare the results with the machine learning models. However, multiple linear regression models were not suitable, due to the parametric conditions were not met.

**Fig. 1:**
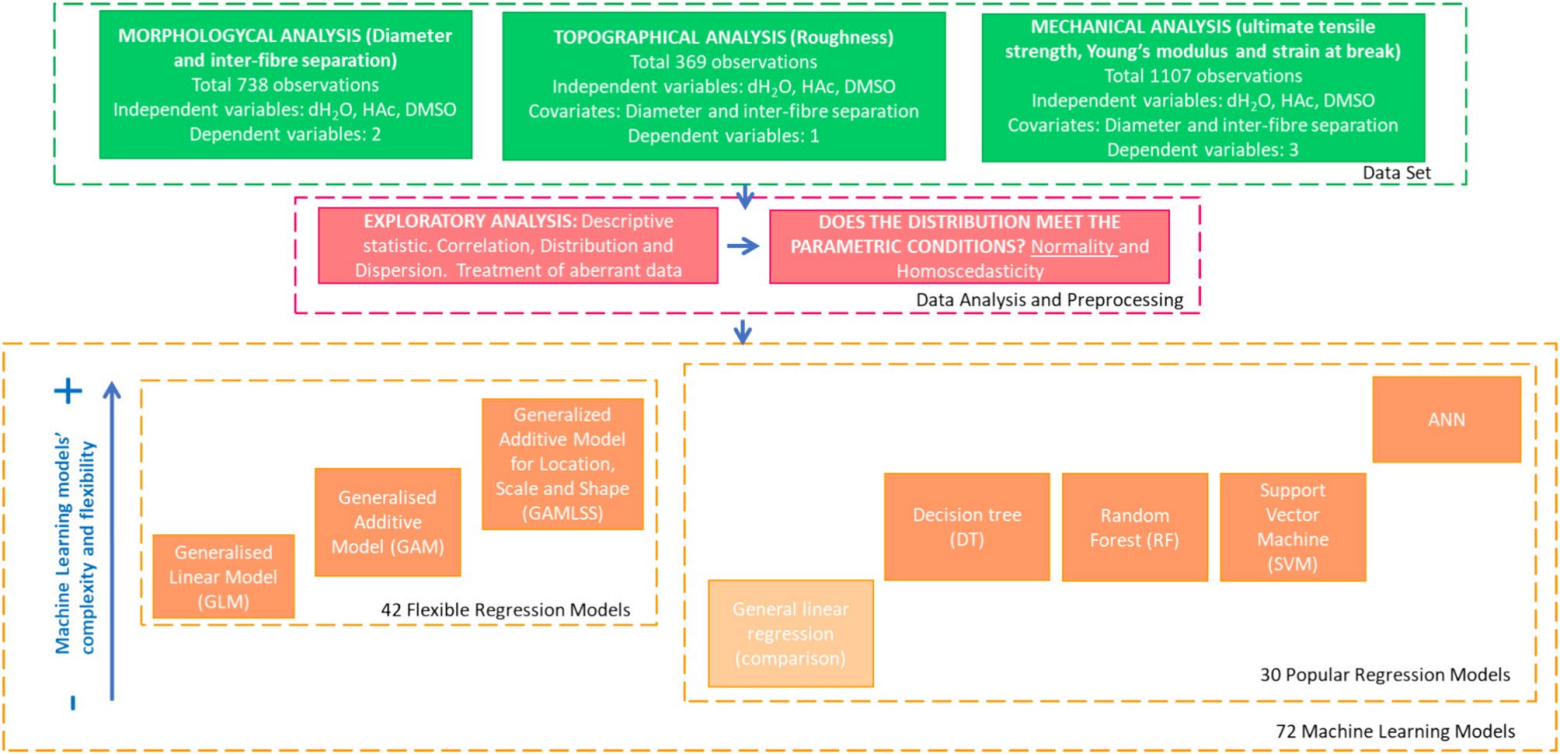
Outline of the followed methodology.

A description of the followed procedure is presented in Fig. 1.

#### Data analysis and preprocessing

An initial exploratory analysis was performed to understand the variables’ distribution, the correlation between variables and treat any aberrant or null data. Continuous variables were used in all of the studied models. In addition, the normality (Kolmogorov Smirnov) and homoscedasticity (Breusch-Pagan) tests were performed to determine if the parametric conditions were met. A total of 2214 observations (369 observations/dependent variable) were analysed.

All initial statistical analyses were conducted using R-4.3.0 and RStudio 2023.03.1, and IBM SPSS v.27 (IBM Inc, US).

#### Flexible regression models: GLM, GAM and GAMLSS

The GLM allows the dependent variable to have a non-normal distribution and follows a distribution from the exponential family (binomial, Poisson, gamma, logistic, etc.). The mean of the predicted variable is related through a link function g(.), following Eqs. (1) and (2), and is no longer directly related to the predictors or independent variables^62^.

1 $$g(\mu )=\beta 0+\beta 1x1+\beta 2x2+...+\beta pxp$$

2 $$g(\mu )=XT\beta$$

The initial statistical analysis proved that none of the response variables followed a normal distribution, and they did not meet the homoscedasticity, the Box test and independence. Therefore, gamma (GA) and Box-Cox-Cole-Green-origin (BCCGo) distributions were assessed for the six dependent variables with a logarithmic function for µ and σ and a RS (Rigby and Stasinopoulos) as fitting method (Table 1).

**Table 1:** Description of Flexible Regression Models.

	Description of the models						
Output variables	Models	Distribution	Parameters GAMLSS				GAM function
			"Mu" µ	"Sigma" σ	"Nu" η	"Tau"τ	
Diameter	lmGD1	NO	Identity	Log	–	–	–
	glmD1	GA	Log	Log	–	–	–
	glmD2	BCCGo	Log	Log	Identity	–	–
	gamD3	BCPEo	Log	Log	Identity	log	Natural splines (ns)
	gamlssD	BCPEo	Log	Log	Identity	log	Natural splines (ns)
	M_psD	LOGNO	Identity	Log	–	–	Natural splines (ns)
	M_nnD	BCPEo	Log	Log	Identity	log	Natural splines (ns)
Inter-fibre Separation	lmGS1	NO	Identity	Log	–	–	–
	glmS1	GA	Log	Log	–	–	–
	glmS2	BCCGo	Log	Log	Identity	–	–
	gamS3	BCPEo	Log	Log	Identity	log	Natural splines (ns)
	gamlssS	BCPEo	Log	Log	Identity	log	Natural splines (ns)
	M_psS	GIG	Log	Log	Identity	–	Natural splines (ns)
	M_nnS	BCPEo	Log	Log	Identity	Log	Natural splines (ns)
Roughness	lmGR1	NO	Identity	Log	–	–	–
	glmR1	GA	Log	Log	–	–	–
	glmR2	BCPEo	Log	Log	Identity	Log	P-splines (pb), natural splines (ns)
	gamR3	IG	Log	Log	–	–	P-splines (pb), natural splines (ns)
	gamlssR	IG	Log	Log	–	–	P-splines (pb), natural splines (ns)
	M_psR	LOGNO	Identity	Log	–	–	P-splines (pb), natural splines (ns)
	M_nnR	IG	Log	Log	Neural network (nn)		P-splines (pb), natural splines (ns)
Ultimate Tensile Strength	lmGT1	NO	Identity	Log	–	–	–
	glmT1	GA	Log	Log	–	–	–
	glmT2	BCPE	Identity	Log	Identity	log	P- splines (pb)
	gamT3	GA	Log	Log		–	P-splines (pb), natural splines (ns)
	gamlssT	GG	Log	Log	Identity	–	P-splines (pb), natural splines (ns)
	M_psT	NO	Identity	Log	Logarithmic transformed		P-splines (pb), natural splines (ns)
	M_nnT	NO	Identity	Log	Neural network (nn)		P-splines (pb), natural splines (ns)
Young's Modulus	lmGY1	NO	Identity	Log	–	–	–
	glmY1	GA	Identity	Log	–	–	–
	glmY2	BCPEo	Log	Log	Identity	Log	P-splines (pb), natural splines (ns)
	gamY3	BCPEo	Log	Log	Identity	Log	P-splines (pb), natural splines (ns)
	gamlssY	BCPE	Identity	Log	Identity	Log	P-splines (pb), natural splines (ns)
	M_psY	NO	Identity	Log	Logarithmic transformed		P-splines (pb), natural splines (ns)
	M_nnY	NO	Identity	Log	Neural network (nn)		P-splines (pb), natural splines (ns)
Strain at break	lmGST1	NO	Identity	Log	–	–	–
	glmST1	GA	Log	Log	–	–	–
	glmST2	BCCGo	Log	Log	Identity	–	P-splines (pb), natural splines (ns)
	gamST3	BCCGo	Log	Log	Identity	–	P-splines (pb), natural splines (ns)
	gamlssST	GA	Log	Log	–	–	P-splines (pb), natural splines (ns)
	M_psST	NO	Identity	Log	Logarithmic transformed		P-splines (pb), natural splines (ns)
	M_nnST	NO	Identity	Log	Neural network (nn)		P-splines (pb), natural splines (ns)

For the GLM the relationship between the predictors and the mean of the dependent variable must be linear and constant. To overcome this limitation the GAMs were introduced. In the GAMs, there is not a direct relationship between predictors xi and the mean of the response variable g(μ) but is done through a function f(xi) (Eq. 3)^62^.

3 $$\eta =g(\mu )=\beta 0+f1(x1)+f2(x2)+...+fp(xp)$$

The function fi(xi) can be linear or non-linear and the most popular functions are non-linear smooth functions such as cubic regression splines, thin plate regression splines or penalised splines (pb). In this study, natural splines (ns) and pb were used.

GAMLSS allows to model the mean (µ, location), variance (σ, scale), skewness (ν, shape) and kurtosis (τ, shape) of the output variables with distributions from the exponential family, based on the predictor variables using linear (X) and non-linear functions (fi(xi))^62^ (Eqs. 4–8).

4 $$Y=XT\beta$$

where Y∼D(μ,σ,ν,τ)

5 $$\eta 1=g1\left(\mu \right)=XT\beta +f1\left(x1\right)+f2\left(x2\right)+\dots +fp\left(xp\right)$$

6 $$\eta 2=g2(\sigma )=XT\beta +f1(x1)+f2(x2)+...+fp(xp)$$

7 $$\eta 3=g3\left(\nu \right)=XT\beta +f1\left(x1\right)+f2\left(x2\right)+\dots +fp\left(xp\right)$$

8 $$\eta 4=g4(\tau )=XT\beta +f1(x1)+f2(x2)+...+fp(xp)$$

In the present study, the function “fitDist()” was used to determine the best-fitted distribution for each output variable. However, sometimes the results did not converge for the best distribution, therefore other kinds of distributions must be evaluated. GA, BCCGo, Box-Cox Power Exponential origin (BCPEo), normal logarithmic (LOGNO), normal (NO), Generalised Inverse Gaussian (GIG), Generalised Gamma (GG) and Inverse Gaussian (IG) distributions have been assessed. Identity and logarithmic was used as the link function for the location (µ), logarithmic was the link function used for the scale (σ) and the identity and the logarithmic were used as link functions for the shape (ν and τ). GAMLSS with transformed logarithmic and neural network were also assessed. A total of 42 GAMLSS models (7 models/dependent variable) were evaluated in this study (Table 1).

To determine the optimum model for each independent variable, six selection criteria were used:

a) The function GAIC(). Where the Akaike information criterion (AIC) values were calculated. AIC is an estimator of the quality of the model that penalised the complex models to avoid overfitting the model, and it is defined for the following Eq. (9).

9 $$AIC = -2 log(L(\theta b)) + 2K$$

where $log(L(\theta b)$ is the logarithmic of the maximum likelihood of the model and K is the number of free parameters of the model.

b) Significance of predictors (Hac, dH_2_O, DMSO and their interactions) for µ, σ, ν and τ.

c) The function wp() (worm plot). Where the residuals must be around the centre of the plot and not invade the elliptical curves which limit the 95% confidence interval.

d) Normality of the residuals (Normal Q-Q plot) and Filliben correlation coefficient (proximal to 1).

e) The function “gamlssCV()” with 10 folds was used for cross-validation.

f) Single validation was performed with the functions “set.seed()” to generate the seed, “sample()” to extract the training sample of 70% of the data, “gamlss()” to train the model and “getTGD()” and “TGD()” to evaluate the model with the test set (30% of the data) and compared between models. Errors and R^2^ were calculated with the test samples for comparison with popular regression models.

All the models were conducted with the “gamlss()” library. The function “drop1()” was used to know the importance of the predictors, and plots were performed with the functions “term.plot()”, “plot.gamlss()” and “centiles()”, this one to plot the centiles. In order to compare between Flexible Regression Models and the rest of the studied regression models, the Mean Absolute Percentage Error (MAPE), Mean Square Error (MSE), Root Mean Square Error (RMSE) and R^2^ were calculated with the library “MLmetrics()”. All these libraries are implemented in R-4.3.0 and RStudio 2023.03.1.

#### Popular regression models

Continuous output variables were used in General Linear Regression, DT, RF, SVM and ANN for regressor purposes. 5 regression models (including linear regression for comparison purposes) were developed for each response variable (a total of 30 models were performed).

All models had a training data sample of 70% (258 observations/dependent variable) and test data of 30% (111 observations/dependent variable).

In order to compare the suitability of the models, the errors MAPE, Mean Absolute Error (MAE), Relative Absolute Error (RAE), MSE, RMSE and the fitted values R^2^ and Gini were calculated with the library “MLmetrics()” and the test data.

Decision trees are a very visual and intuitive prediction technique that determines the importance of the exogenous variables in the endogenous variables. In this study, we used regression decision trees to predict the diameter of the fibre, inter-fibre separation, roughness, ultimate tensile strength, Young’s modulus and strain at break, and determine the importance between those and the solvents’ concentration. 2214 observations of morphology, topography and mechanical properties were used to inform the model. Decision trees with the “anova” method were performed for each independent variable with the libraries of R “rpart()” and “rpart.plot()”. The function “prune()” was applied to find out the essential number of nodes.

The randomForest() package with 1000 trees, replace, vector type (for continuous variables) and 3 variables tried at each split was used to conduct RF models for each dependent variable and calculate the importance of the independent variables.

For the regression SVM models, the SVM type was nu-regression, the SVM Kernel was polynomial, nu was 0.5, and the cost was defined as 1. SVM models were validated through cross-validation with 3 sample folds. The library “e1071()” of R-4.3.0 was used for this study.

The “neuralnet()” library was used for its efficiency and ease of use to predict the 6 output variables. In all models, the algorithm used was the resilient backpropagation with weight backtracking, 2 hidden layers and 3 neurons in each layer. The activation function used for all units in the hidden layers and output layer was the logistic. The rest parameters were selected as default in the “neuralnet()” library.

All models were conducted with packages and libraries implemented in R-4.3.0 and RStudio 2023.03.1.

## Results

### Data analysis and preprocessing

An initial exploratory analysis was performed to understand the variables’ distribution, the correlation between variables and to assess aberrant or null data, this can be found in the supplementary material. A concise summary of the descriptive statistics done during the exploratory analysis is presented in Table 2. After performing the normality (Kolmogorov Smirnov test) and homoscedasticity (Breusch-Pagan test) tests, it was proved that none of the 6 output variables followed a normal distribution (*P* value < 0.001), and they did not meet the homoscedasticity (*P* value < 0.001). Therefore, popular statistics models such as general linear models or Multivariate Analysis of Variance (MANOVA) models were not suitable for this study, and the suitability of 12 machine learning techniques was evaluated. All the exploratory analyses and tests can be found in the supplementary material.

**Table 2:** Descriptive Statistics.

		Diameter	Separation	Roughness	Tensile	Young's Modulus	Strain at break
N	Valid	369	369	369	369	369	369
	Lost	0	0	0	0	0	0
Mean		0.4583	2.0584	1.2415	3.1972	223.2405	1.6459
Standard Error		0.0183	0.1	0.02	0.077	4.5526	0.0355
Standard Deviation		0.3514	1.9212	0.3843	1.479	87.4532	0.6822
Variance		0.1235	3.6909	0.1477	2.1876	7648.067	0.4653
Skewness		1.3402	2.1822	0.341	0.0267	0.0172	0.0302
Kurtosis		1.5174	6.413	− 0.8329	− 1.5605	− 1.0247	− 0.4188
Percentile	10	0.1426	0.4364	0.7703	1.3877	107.1931	0.8538
	50	0.32	1.4	1.2326	3.5984	221.7331	1.7114
	90	0.956	4.592	1.7479	5.0172	326.7112	2.6673

### Flexible regression models: GLM, GAM and GAMLSS

The gold standard for flexible regression models is the Akaike information criterion (AIC). We adopted this criterion, in combination with the other criteria explained in section "Flexible regression models: GLM, GAM and GAMLSS", to determine the best flexible model.

**Fig. 2:**
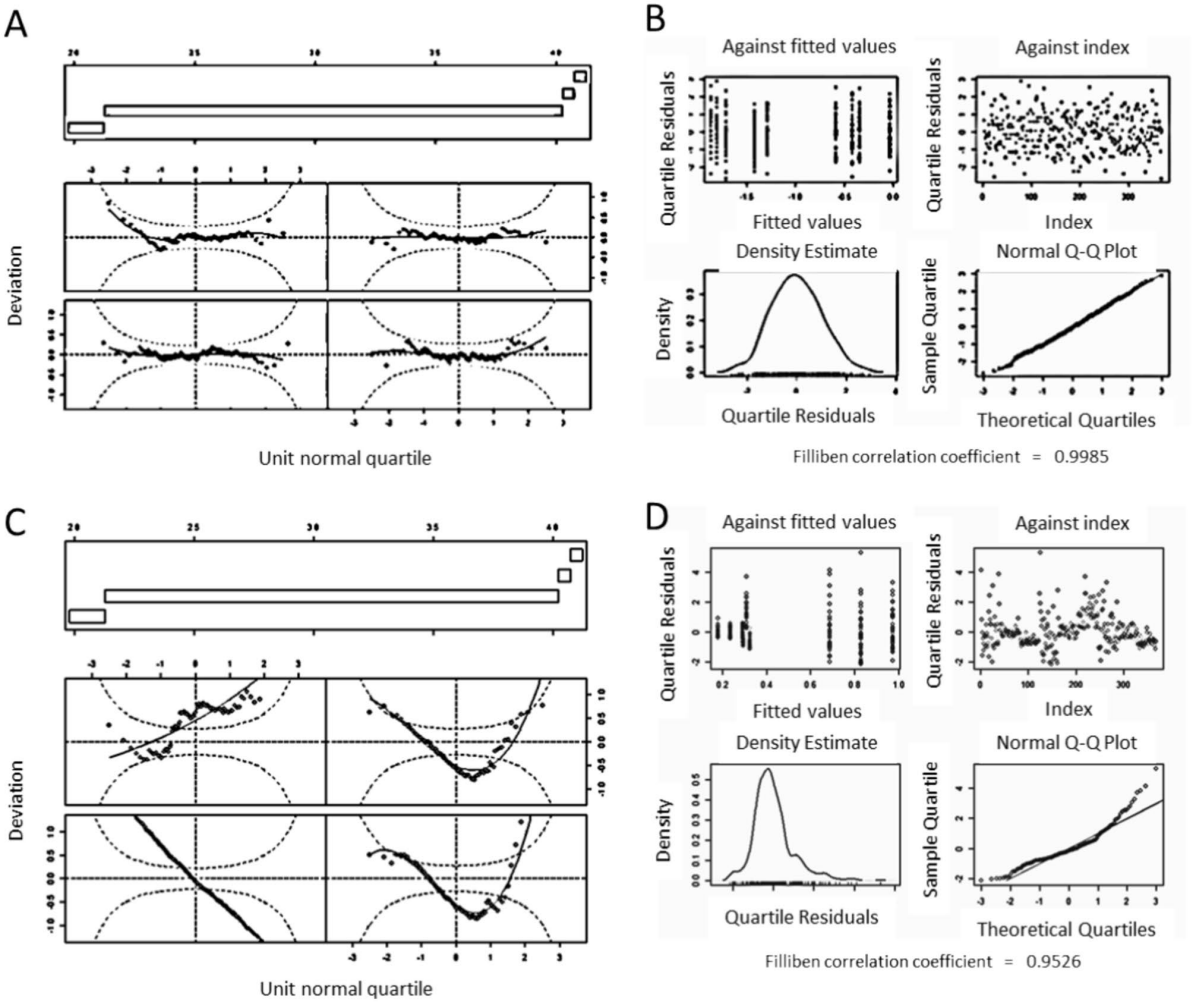
(**A**) Good example of worm plot (M_psD), (**B**) Good example of plot.gamlss residuals’ distribution (M_psD), (**C**) Bad example of worm plot (lmGD1), (**D**) Bad example of plot.gamlss residuals’ distribution (lmGD1).

The model which best represents the observed values of the diameter of the fibres was the “M_psD”, following AIC (Table 3). The worm plots showed that the distribution of the residuals was centred and they did not invade the elliptical curves (confidence interval 95%) (Fig. 2A). The plot.gamlss showed that the distribution of the residuals followed a normal distribution and it was also proved with a Filliben’s coefficient of 0.9998 (Fig. 2B). Moreover, R^2^ (0.705) and RMSE (0.1904), calculated with test data, proved that the model “M_psD” was the best for the diameter (Table 3). To the contrary, and as it was expected, the general linear regression (lmGD1) was the one that provided the worst fit in diameters. The residuals for “lmGD1” were not centred, and more than 50% of them were out of the 95% confidence interval (Fig. 2C). Moreover, the residuals did not follow the normal distribution (Fig. 2D), and Filliben’s coefficient was the lowest (0.9526).

**Table 3:** Model selection, errors and R^2^.

Output variables	Models	Model selection following AIC and residuals performance				Regression model comparison			
		AIC	Worm plot		Filliben	Errors			R^2^
			Centred residuals (Y/N)	% Out of range		MAPE	MSE	RMSE	
Diameter	lmGD1	− 59.67	N	> 15	0.9526	0.4402	0.0611	0.2189	0.61
	glmD1	− 278.61	N	> 15	0.990334	0.4068	0.0479	0.2189	0.6105
	glmD2	− 354.78	N	> 15	0.99589	0.3139	0.0534	0.2311	0.5661
	gamD3	− 592.72	Y	0	0.9991	0.2397	0.0369	0.1923	0.6996
	gamlssD	− 598.04	Y	0	0.9989	0.2427	0.0367	0.1917	0.7015
	M_psD	− 606.32	Y	0	0.9985	0.2429	0.0363	0.1904	0.705
	M_nnD	− 246.61	Y	0	0.9991	0.2399	0.0368	0.1923	0.6995
Inter-fibre Separation	lmGS1	1342.94	N	> 15	0.93011	0.7368	2.1459	1.4649	0.417
	glmS1	985.60	Y	< 5	0.996326	0.7356	2.1531	1.4673	0.415
	glmS2	985.65	Y	< 5	0.99714	0.7329	2.1533	1.4674	0.4149
	gamS3	940.75	Y	0	0.998	0.6184	2.1632	1.4708	0.4123
	gamlssS	943.97	Y	0	0.9966	0.6184	2.16	1.4707	0.4129
	M_psS	953.93	Y	0	0.932	0.6925	2.0393	1.428	0.4459
	M_nnS	1288.94	Y	0	0.9979	0.6291	2.0858	1.4442	0.6995
Roughness	lmGR1	28.65	N	> 15	0.9855102	0.1735	0.0602	0.2454	0.5907
	glmR1	5.06	N	0	0.981034	0.1707	0.059	0.2431	0.5984
	glmR2	− 5.90	Y	0	0.989597	0.1675	0.063	0.2511	0.5715
	gamR3	− 86.34	N	< 15	0.9713	0.1288	0.0386	0.1965	0.7377
	gamlssR	− 331.10	N	0	0.9854	0.1298	0.0401	0.2003	0.7274
	M_psR	− 319.3	Y	0	0.98797	0.1261	0.0389	0.1972	0.7354
	M_nnR	126.30	N	0	0.97739	0.1101	0.0285	0.1688	0.8064
Ultimate Tensile Strength	lmGT1	1047.84	N	> 15	0.9459	0.2908	0.9504	0.9767	0.5626
	glmT1	1061.32	N	< 10	0.973635	0.3044	1.1874	1.0897	0.4557
	glmT2	883.99	N	< 10	0.99517	0.2024	0.9768	0.9883	0.5522
	gamT3	504.19	N	> 15	0.976	0.141	0.2725	0.522	0.875
	gamlssT	358.66	N	< 5	0.9814	0.165	0.3787	0.6154	0.8264
	M_psT	-293.40	N	< 5	0.9685	0.141	0.2752	0.5246	0.8738
	M_nnT	42.70	N	0	0.9896	0.0949	0.147	0.3835	0.9326
Young's Modulus	lmGY1	4279.51	N	> 15	0.9676	0.3543	6068.9	77.903	0.2043
	glmY1	4288.94	N	> 15	0.99026	0.3608	6463.2	80.394	0.1526
	glmY2	3478.87	N	< 5	0.990564	0.1345	678.29	26.044	0.911
	gamY3	3477.05	N	< 5	0.9905	0.1345	678.29	26.04	0.911
	gamlssY	3269.56	N	< 5	0.9848	0.1301	649.34	25.48	0.9148
	M_psY	− 652.33	N	0	0.9848	0.1108	550.21	23.457	0.9278
	M_nnY	− 3.73	N	> 15	0.9269	0.0662	258.57	16.08	0.966
Strain at break	lmGST1	454.76	N	> 15	0.99131	0.4343	0.1912	0.4373	0.5879
	glmST1	610.66	N	< 5	0.961599	0.431	0.211	0.4594	0.5451
	glmST2	485.82	N	< 5	0.9915553	0.5177	0.1989	0.446	0.5713
	gamST3	374.10	N	> 15	0.9783	0.515	0.1827	0.4274	0.6063
	gamlssST	75.80	N	> 15	0.979	0.4844	0.2203	0.4694	0.5251
	M_psST	− 191.08	N	< 5	0.9814	0.3129	0.1575	0.3969	0.6605
	M_nnST	684.68	N	> 15	0.9915	0.2747	0.1443	0.3798	0.689

Following the AIC, the model “gamS3” represented the best fit for the inter-fibre separation. Its residuals were centred, did not invade the elliptical curves, followed a normal distribution, and the Filliben’s coefficient was excellent (0.998). Moreover, this model provided the best MAPE (Table 3).

The best prediction model for roughness was the “gamlssR”, following the AIC. However, “M_psR” provided AIC values very similar, and its residuals, errors and R^2^ behaved better than the model “gamlssR”. The model that provided the best errors and R^2^ for test data was the “M_nnR” with a MAPE of 0.1101 and R^2^ of 0.8064 (Table 3); however, AIC penalised that model for its complexity and possible overfitting.

“M_psT” was the best model to predict the ultimate tensile strength with AIC value much lower than the values of the other models. However, the residual of the “M_nnT” model behaved better than “M_psT”, and the errors (MAPE = 0.0949) and R^2^ (0.9326) were the best of all the models. Moreover, the AIC of “M_nnT” was the second lowest, which indicates that this model should be also considered.

“M_psY” was the best prediction model for Young’s modulus, following AIC and cross-validation method. The residuals were not out of the 95% confidence interval; however, they were not horizontally centred. The model “M_nnY” provided the second lowest AIC and gave excellent errors (MAPE = 0.0662) and R^2^ (0.966); however, the residuals behaved worse than the residuals of the model “M_psY”.

The best prediction model for the strain at break was “M_psST” following AIC and cross-validation. Its worm plot showed non-centred residuals, and < 5% of the residuals invaded the elliptical curves. The best errors and R^2^ were provided by the “M_nnST” model; however, its residuals were not centred and invaded > 15% of the curves; moreover, this model was the most complex and could be overfitted.

Once the best flexible models are determined for each dependent variable, the significance of the coefficients of the predictors and interactions were evaluated. Table 4 showed that most of the regression coefficients of µ for “M_psD”, “gamlssR”, “M_spT”, “M_spY” and “M_spST” were highly significant.

**Table 4:** Best flexible models of each output variables (following AIC): Coefficients and their significance.

Output Variables	Models	Distribution	Parameters	Link	Coefficients										
					Intercept	pb(Diameter)	pb(Separation)	HAc	dH_2_O	DMSO 1	DMSO 2	HAc x DMSO 1	HAc x DMSO 2	dH_2_OxDMSO 1	dH_2_OxDMSO 2
Diameter	M_psD	LOGNO	µ	Identity	− 1.8033			1.733	− 0.0713	0.795	0.3153	− 0.6581	0.0732	1.499	− 1.264
					***			***		***	***	**		***	***
			σ	Log	− 1.2525			0.3864	− 0.255	− 0.262	− 0.004	0.2016	− 0.633	0.733	0.29
					***			*					*		
Inter-fibre Separation	gamS3	BCPEo	µ	log	− 0.3937			1.7713	0.0975	0.9206	0.1579	− 0.7228	0.41	0.917	− 0.283
					***			***		***				*	
			σ	Log	− 0.6702			0.2432	0.1048	0.0245	− 0.0913	− 0.3904	0.4163	0.2912	− 0.1462
					***								*		
			η	Identity	0.3225			− 0.4976	− 0.0913	0.3678	0.2391	1.1132	− 0.5654	0.2888	0.0317
			τ	Log	1.2355										
					***										
Roughness	gamlssR	IG	µ	log	0.2906	− 0.013	− 0.01239	0.2611	− 0.0253	− 0.9225	− 0.165	0.8604	0.6386	0.8526	− 0.0522
					***		***	***		***	***	***	***	***	
			σ	Log	− 1.8178	0.3352	0.1248	− 1.9579	− 0.6139	− 2.605	1.2502	3.802	− 1.9743	4.2724	− 1.2145
					***		***	***	**	***	***	***	***	***	***
Ultimate Tensile Strength	M_spT	NO	µ	Identity	1.539	0.0231	− 0.0107	− 1.1415	− 0.02778	− 0.3292	− 0.969	1.6456	0.4052	− 0.1745	− 0.4959
					***			***		***	***	***	***		***
			σ	Log	1.9339	0.7725	− 0.008	0.5865	− 0.8265	0.2482	0.103	0.108	− 0.4416	1.6212	0.4744
					***			*	***					**	
Young's Modulus	M_spY	NO	µ	Identity	5.336	− 0.03	0.002	− 0.69	0.273	0.1709	− 1.04	1.871	0.531	− 0.22	0.699
					***	**		***	***	***	***	***	***	***	***
			σ	Log	− 2.4262	− 0.049	− 0.066	0.2391	− 0.3116	− 0.0436	1.8306	0.1505	− 0.9865	− 1.9135	− 1.679
					***						***		***	***	***
Strain at break	M_spST	NO	µ	Identity	0.8909	0.00417	0.0092	− 0.2457	− 0.2964	− 0.3582	− 0.6441	− 0.7948	0.721	− 0.6134	− 1.023
					***			***	***	***	***	***	***	***	***
			σ	Log	− 1.9799	− 0.2386	0.1333	0.8761	− 2.1821	− 1.0718	1.2207	− 1.9437	− 2.3131	7.6277	0.843
					***		*	**	***	***	***	***	***	***	*

*P value* = 0 ‘***’, 0 < *P value* < 0.001 ‘**’, 0.001 < *P value* < 0.01 ‘*’, 0.1 < *P value* < *0.05 ‘.’, 0.05* < *P value* < *0.1 ‘ ’.*

**Fig. 3:**
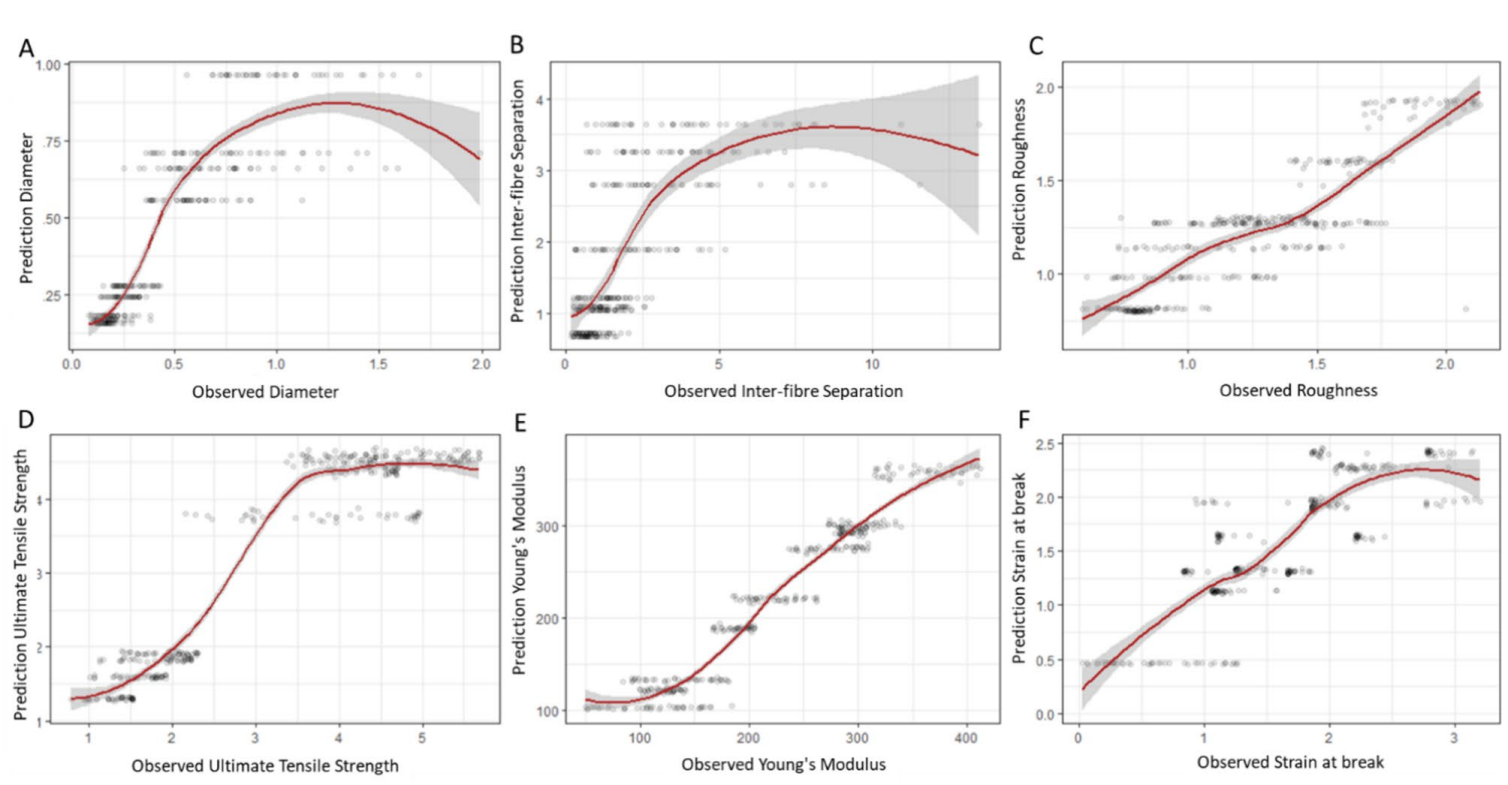
Prediction vs observed values of the µ of each response variable generated with the best models following AIC. (**A**) Diameter (µm), (**B**) Inter-fibre Separation (µm), (**C**) Roughness (µm), (**D**) Ultimate Tensile Strength (MPa), (**E**) Young’s Modulus (MPa), (**F**) Strain at break (%).

The observed and predicted values for each dependent variable were shown in Fig. 2. The behaviour of the mechanical and topographical properties was comparable, exhibiting smoothed functions with very small confidence intervals and gradients close to 1. However, observations of morphology (Diameter of the fibres and Inter-fibre Separation) with a percentile superior to 95% generated a negative gradient and a severe increase in the confidence interval. Figure 3 shows the goodness of fit of the selected models.

After the validation of the models, the effects of the solvents and covariates on the response variables were studied for location (µ), scale (σ) and shape (ν and τ). The function “drop 1” allowed us to know the total contribution (linear and non-linear) of the smoothed predictors on the output variables.

**Fig. 4:**
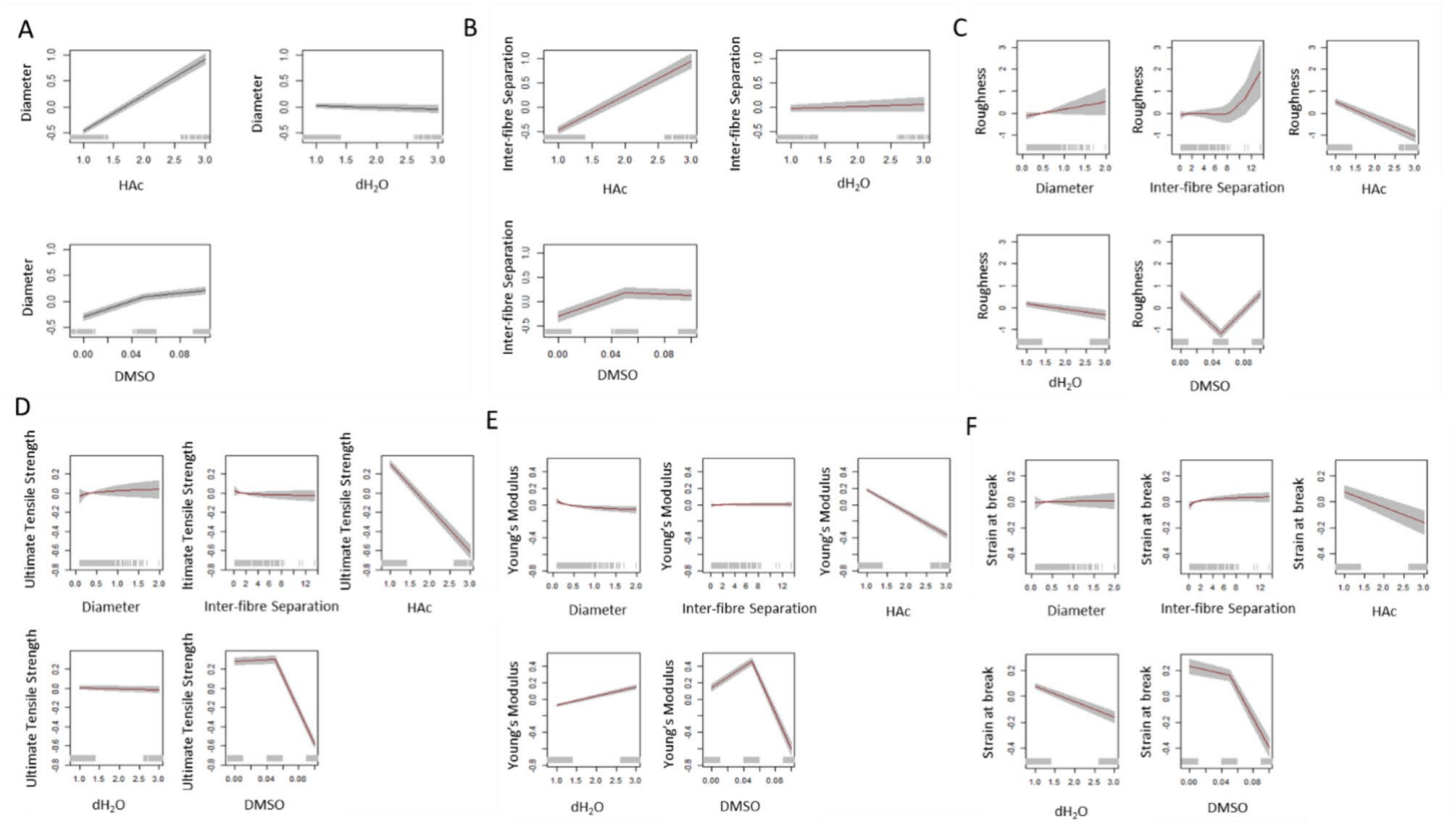
Effect of the concentration of the solvents and covariates on the µ of response variables for each best model following AIC (**A**) Diameter, (**B**) Inter-fibre Separation, (**C**) Roughness, (**D**) Ultimate Tensile Strength, (**E**) Young’s Modulus and (**F**) Strain at break.

Analysing the impact of the solvents’ concentration on the µ of the diameter of the fibres for the “M_psD” (Fig. 4A), we observed that the HAc had a positive linear behaviour (as more HAc concentration, more diameter of the fibres). The influence of the H_2_O on the diameter of the fibres was negligible. And the contribution of DMSO had a positive slope but with different gradients depending on the DMSO’s levels.

Similar effects were observed for the µ of the inter-fibre separation with the model “gamS3” (Fig. 4B). The HAc increased when the inter-fibre separation increased, the H_2_O did not contribute to the inter-fibre separation and the DMSO experienced a positive gradient at low concentrations and did not contribute to higher concentrations.

The model “gamlssR” included two covariates (Diameter of the fibres and Inter-fibre Separation) to the solvents’ concentrations to predict the µ of the roughness (Fig. 4C). The diameter had a positive linear behaviour on the roughness. The effect of the inter-fibre separation on the roughness was negligible when its values were small; however, when the values of inter-fibre separation increased the roughness exponentially increased. The HAc negatively influences the roughness. The H_2_O had a minimum influence. And the DMSO showed a V-shape on the roughness.

The covariates (Diameter and Inter-fibre Separation) did not have any effect on the µ of the Ultimate Tensile Strength (Fig. 4D). The HAc had a negative effect, the H_2_O did not exhibit any effect, and the DMSO had not any effect with low concentrations but had a negative impact on the Ultimate Tensile Strength for high concentrations.

The effects of the covariates and concentration of HAc were similar on the µ of the Ultimate Tensile Strength, Young’s Modulus (Fig. 4E) and Strain at break (Fig. 4F). However, the H_2_O experienced a negative impact on the µ of Young’s Modulus and a positive influence on the µ of the Strain at break. Low concentration of DMSO had a positive impact on µ of the Young’s Modulus and a negative effect on the µ of the Strain at break.

The coefficients of the solvents’ concentrations and covariates on the σ, η and τ of the response variables were not significant (Table 3), therefore they were not considered in this study.

### Popular Regression Models

Errors and goodness of fit were calculated with test data and compared with general linear model (LM), DT, RF, SVM and ANN (Table 5). The worst model to predict the diameter of the fibres was the LM with an R^2^ of 0.4846 and RMSE of 0.2706, and the best fit was with the SVM with an R^2^ of 0.6378 and RMSE of 0.2392. In terms of Inter-fibre Separation, the worst models were the LM and the SVM, on the contrary, the best models were the ANN and the RF. The worst model to predict roughness was DT, and the best behaviour was obtained with ANN. Regarding the Ultimate Tensile Strength the worst model was the LM, and the best one was RF with an R^2^ of 0.8486 and MAPE of 0.1496. ML exhibited the worst behaviour for Young’s modulus, and the best two models were the ANN and the DT with an R^2^ of 0.9294 and 0.9281 respectively and MAPE of 0.13.30 and 0.1032. The two worst models to predict Strain at break were DT and SVM, and the two best models were ANN and RF.

**Table 5:** Regression models comparison.

Output Variables	Models	Errors					R^2^	Gini
		MAPE	MAE	RAE	MSE	RMSE		
Diameter	LM	0.4493	0.1766	0.6118	0.0732	0.2706	0.4846	0.7667
	DT	0.2780	0.1307	0.4528	0.0542	0.2327	0.6219	0.8665
	RF	0.2789	0.1398	0.4536	0.0541	0.2327	0.6190	0.8665
	SVM	–	–	–	0.0480	0.2392	0.6378	–
	ANN	0.3066	0.1361	0.4713	0.0556	0.2358	0.6084	0.7667
Inter-fibre Separation	LM	0.7890	0.8246	0.7102	1.4775	1.2156	0.3995	0.6942
	DT	0.6713	0.7460	0.6423	1.3084	1.1441	0.4680	0.7530
	RF	0.6701	0.7451	0.6416	1.3050	1.1427	0.4693	0.7591
	SVM	–	–	–	2.4066	1.5513	0.3960	–
	ANN	0.6726	0.7454	0.6419	1.3026	1.1413	0.4706	0.6942
Roughness	LM	0.1849	0.1995	0.6728	0.0553	0.2351	0.5442	0.7659
	DT	0.1927	0.2020	0.6829	0.0700	0.2653	0.4194	0.6660
	RF	0.1847	0.1952	0.6580	0.0610	0.2469	0.4968	0.6930
	SVM	–	–	–	0.0660	0.2569	0.5500	–
	ANN	0.1594	0.1726	0.5824	0.0474	0.2177	0.6093	0.7659
Ultimate Tensile Strength	LM	0.3024	0.7976	0.6002	0.9030	0.9503	0.5749	0.7770
	DT	0.1663	0.4786	0.3601	0.3790	0.6156	0.8216	0.8160
	RF	0.1496	0.4323	0.3253	0.3214	0.5670	0.8486	0.8627
	SVM	–	–	–	0.7167	0.8466	0.6800	–
	ANN	0.1627	0.4552	0.3425	0.3523	0.5936	0.8341	0.8800
Young's Modulus	LM	0.6123	61.2300	0.8260	5,971.0000	77.2700	0.1889	0.3342
	DT	0.1032	18.9300	0.2475	529.9000	23.0010	0.9281	0.9553
	RF	0.1191	20.2300	0.2730	677.0000	26.0200	0.9079	0.9466
	SVM	–	–	–	521.3000	72.2000	0.3152	–
	ANN	0.1330	18.4300	0.2486	519.1400	22.7800	0.9294	0.9589
Strain at break	LM	0.3374	0.3244	0.6071	0.1653	0.4066	0.6310	0.7886
	DT	0.3419	0.3880	0.5778	0.1500	0.3874	0.6651	0.8224
	RF	0.3367	0.2925	0.5474	0.1384	0.3720	0.6912	0.8426
	SVM	–	–	–	0.2142	0.4629	0.5256	–
	ANN	0.3283	0.3041	0.5690	0.1549	0.3935	0.6545	0.8092

**Fig. 5:**
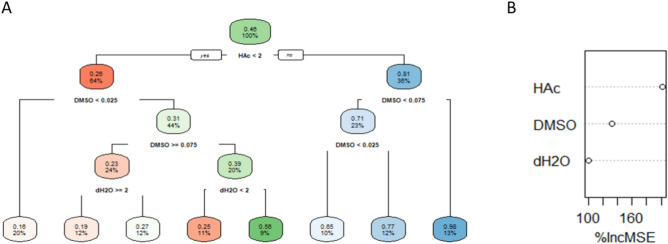
Visualization of (**A**) DT and (**B**) RF to predict the Diameter.

Figure 5 represents the importance of the predictors Hac, dH_2_O and DMSO on the variable Diameter. The importance of each predictor was evaluated with DT and RF corroborating the results provided in Fig. 3. A full presentation of the results and the importance given by those methods was included in the support material.

## Discussion

This research provides a novel and systematic methodology to compare flexible regression models (GLM, GAM and GAMLSS) and popular regression models (DT, RF, SVM and ANN).

There are models easy to interpret (e.g. DT) and others whose interpretation is more complicated, although they often provide better goodness of fit (e.g. ANN)^39^. To compare within models, different errors and fit indexes were calculated. R^2^ was used in this research due to its popularity in the scientific community. The MAPE (Mean Absolute Percentage Error) was selected due to its ease of interpretation and allows comparison between the dependent variables. The best MAPE and R^2^ of the flexible regression models and the best of the popular regression models were compared for each one of the six response variables. The R^2^ was improved with the best GAMLSS models by 6.868%. The highest increment was observed with the Inter-fibre Separation with an increment of 35%. On the contrary, the Strain at break did not exhibit a remarkable increment. Regarding MAPE, the best GAMLSS models improved the MAPE performance by 21.16% with respect to the popular regression models. The MAPE improvement range was between 8.3% for the Inter-fibre Separation and 57.64% of improvement for the Ultimate Tensile Strength.

ANN models provided the best fit of the popular regression models studied for three of the dependent variables (Inter-fibre Separation, Roughness and Young’s Modulus). Linear regressions obtained the highest MAPE in four of the variables (Diameter, Inter-fibre Separation, Ultimate Tensile Strength and Young’s Modulus), given the lowest accuracy for those variables. This fact was corroborated also in different studies^38,39,63^. RF models were more accurate than DT in four output variables due to these models calculated 1000 trees which improved the performance and stabilise the prediction, a fact also observed in previous studies^52^.

Regarding the flexible regression models, the majority of the models created with a logarithmic scale for the covariates and the output variable (“M_ps”) provided the lowest AIC, demonstrating better goodness of fit than other models, without increasing the complexity of the model and avoiding overfitting. The GAMLSS models created with the “gamlss.add()” library, which include the multilayer perceptron algorithm (“M_nn”), obtained the best R^2^, MAPE, MSE and RMSE in four of the response variables. However, the AIC penalised their complexity which could induce overfitting.

One of the objectives of this study is to find the models that best predict the Diameter of the fibres, Inter-fibre Separation, Roughness, Ultimate Tensile Strength, Young’s modulus and Strain at break; and therefore, to know how the green solvents’ concentration behaves with the output variables.

The importance of the input variables calculated with GAMLSS coincided with the ones calculated by DT, RF and ANN. Popular regression models proved that the HAc has a high level of importance in the formation of the morphological (> 75%, see supplementary material and the topographical variables. Flexible regression models (Fig. 3A) showed the positive gradient of the Diameter and Inter-fibre Separation with respect to the increment of HAc without considering the interactions between the solvents. The pruned DT revealed that the morphological properties increased with the increment of HAc and DMSO. This was also verified by Erencia et al^19^. who proved that an increment of HAc concentration provokes an increment in the diameter of the fibres.

Moderated concentrations of DMSO (5%) highly influence the variation of the mechanical properties. The term plots from GAMLSS exhibited that DMSO between 5 and 10% negatively influenced the behaviour of the three mechanical properties (Fig. 3D, E and F). The pruned DT for the mechanical properties confirmed this conclusion. The highest Ultimate Tensile Strength were obtained with concentrations of DMSO of 5% and HAc of 1:3. The highest Young’s Modulus was achieved with concentrations of DMSO of 5%. And the highest Strain at break was produced with a concentration of DMSO of 5% and diameters of the fibre between 0.32 and 0.17 µm and interfibre-separation between 1.5 and 0.98 µm. These values were observed with the complete DT.

The DT showed that the roughness increased with the concentration of HAc and with high levels of DMSO. However, the term plots from GAMLSS (Fig. 3C) showed that the HAc considered individually (without interactions with the other solvents) had a negative effect the roughness, and the DMSO showed a “V shape”.

The term plot, DT and RF showed that the H_2_O did not influence the morphology, topography and mechanical properties. However, we believe that its influence is important as a coadjuvant in the dissolution, due to the concentration of HAc highly depends on the concentration of H_2_O and vice versa.

The use of the morphological properties as covariates in the roughness and mechanical properties allowed us to understand the behaviour of the morphological properties in the rest of the properties. The diameter of the fibre between 0.32 µm and 0.17 (20 and 50 percentiles) with an inter-fibre separation between 1.5 and 0.98 µm (30 and 50 percentiles) provided a higher strain at break. The highest ultimate tensile strength was obtained with scaffolds with diameter of fibres above 0.16 µm (20 percentile) and inter-fibre separation between 0.82 and 0.61 µm (percentiles 30 and 20). Regarding Young’s modulus, values of diameter between 0.24 and 0.82 µm (percentiles 40 and 80) generated the highest Young’s modulus.

Introducing the morphological properties as covariates for the prediction of roughness, we were able to conclude that values of inter-fibre separation below 0.7 µm (20 percentile) generated the highest roughness. However, values of diameter of the fibres lower than 0.36 µm (50 percentile) provoked the smallest roughness.

## Conclusions

In this research, twelve different machine learning techniques, including flexible regression models and popular regression models, were compared to determine the best models to predict the influence of green solvents on the morphology, topography and mechanical properties of gelatin-based scaffolds.

It was observed that the best GAMLSS models (with the lowest AIC) exhibited better goodness of fit (R^2^) than the popular regression models, with an increment of R^2^ of 6.868%. The accuracy of these models was also higher than popular models with an increment of MAPE of 21.16%. Between the flexible models, the ones created with a transformed logarithmic for the covariates and the output variable (“M_ps”) provided better goodness of fit than other models, without increasing their complexity and avoiding overfitting.

Regarding the influence of the concentration of the green solvents on the morphology, topography and mechanical properties, it was observed that HAc highly affected the morphology and topography of the scaffolds; however, the importance of DMSO was more relevant than the other solvents in the mechanical properties of the scaffolds. Moreover, the inclusion of the morphological properties as covariates in the topographic and mechanical models allowed a better understanding of them.

## Supplementary Information

Supplementary Information.

## Data Availability

The data supporting this article will be made available on request to the correspondence author Elisa.Roldan-Ciudad@mmu.ac.uk.
