# Supplementary material for: Machine learning to predict morphology, topography and mechanical properties of sustainable gelatin-based electrospun scaffolds
Source: Sci Rep. 2024 Sep 9;14:21017. doi: 10.1038/s41598-024-71824-2 (PMC11385233; doi:10.1038/s41598-024-71824-2)
Supplement: Supplementary file 1 — Supplementary Information. [file 41598_2024_71824_MOESM1_ESM.pdf]

Supplementary material for the article:

Machine learning to predict morphology, topography and mechanical properties of sustainable gelatin-based electrospun scaffolds

Elisa Roldán[1]*, Neil Reeves[2], Glen Cooper[3], Kirstie Andrews[1]

[1] Department of Engineering, Faculty of Science & Engineering, Manchester Metropolitan University, Manchester M1 5GD, UK

[2] Research Centre for Musculoskeletal Science & Sports Medicine, Department of Life Sciences, Faculty of Science & Engineering, Manchester Metropolitan University, Manchester M1 5GD, UK

[3] School of Engineering, University of Manchester, Manchester M13 9PL, UK

*** Correspondence:**
Elisa.Roldan-Ciudad@mmu.ac.uk

1 Exploratory Analysis

Supplementary Material Table 1. Exploratory Analysis

		Diameter	Separation	Roughness	Tensile	Young	Strain
N	Valid	369	369	369	369	369	369
	Lost	0	0	0	0	0	0
Mean		,4583	2,0584	1,2415	3,1972	223,2405	1,6459
Standard Error		,0183	,1000	,0200	,0770	4,5526	,0355
Median		,3200[a]	1,4000[a]	1,2326[a]	3,5984[a]	221,7331[a]	1,7114[a]
Mode		,1600	,4300	,8099	,7777[c]	50,3718[c]	,0207[c]
Standard Deviation		,3514	1,9212	,3843	1,4790	87,4532	,6822
Variance		,1235	3,6909	,1477	2,1876	7648,0670	,4653
Skewness		1,3402	2,1822	,3410	,0267	,0172	,0302
Skewness Standard Error		,1270	,1270	,1270	,1270	,1270	,1270
Kurtosis		1,5174	6,4130	-,8329	-1,5605	-1,0247	-,4188
Kurtosis Standard Error		,2533	,2533	,2533	,2533	,2533	,2533
Range		1,9100	13,3000	1,5424	4,9010	361,3249	3,1821
Minimum		,0800	,1800	,5896	,7777	50,3718	,0207
Percentile	10	,1426[b]	,4364[b]	,7703[b]	1,3877[b]	107,1931[b]	,8538[b]
	20	,1710	,6777	,8313	1,5782	131,1093	1,0841
	30	,2039	,9123	,9376	1,9164	164,5405	1,1675
	40	,2492	1,1379	1,1146	2,1775	191,6881	1,3010
	50	,3200	1,4000	1,2326	3,5984	221,7331	1,7114
	60	,4216	1,7580	1,3345	4,0416	267,1753	1,8664
	70	,5782	2,1487	1,4596	4,3743	287,5993	1,9818
	80	,7557	3,3170	1,6084	4,6709	302,1032	2,2074
	90	,9560	4,5920	1,7479	5,0172	326,7112	2,6673

Supplementary Material Table 2. Kolmogorov Smirnov Test

	Statistic	DoF	P value
Diameter	,164	369	<,001
Separation	,183	369	<,001
Roughness	,093	369	<,001
Tensile	,177	369	<,001
Young	,112	369	<,001
Strain	,100	369	<,001

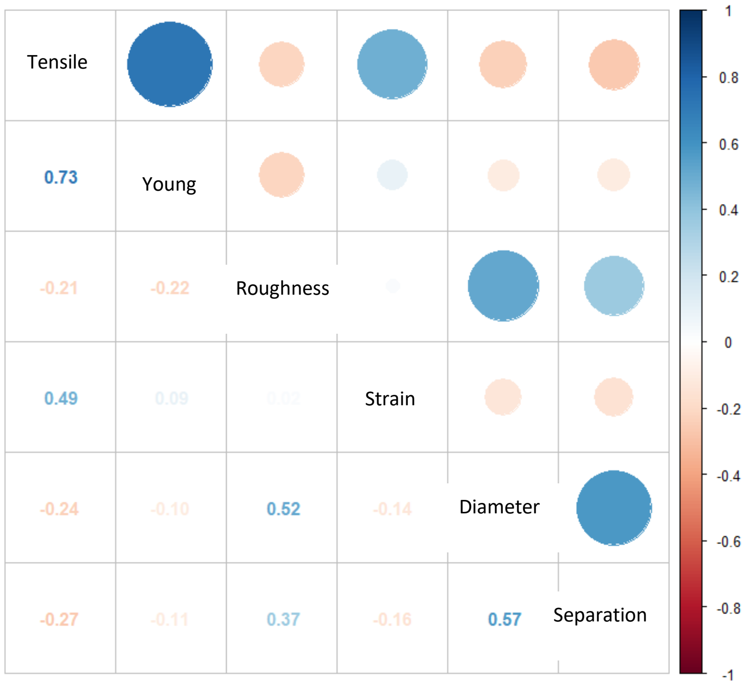

Supplementary Material Figure 1. Correlation between dependent variables

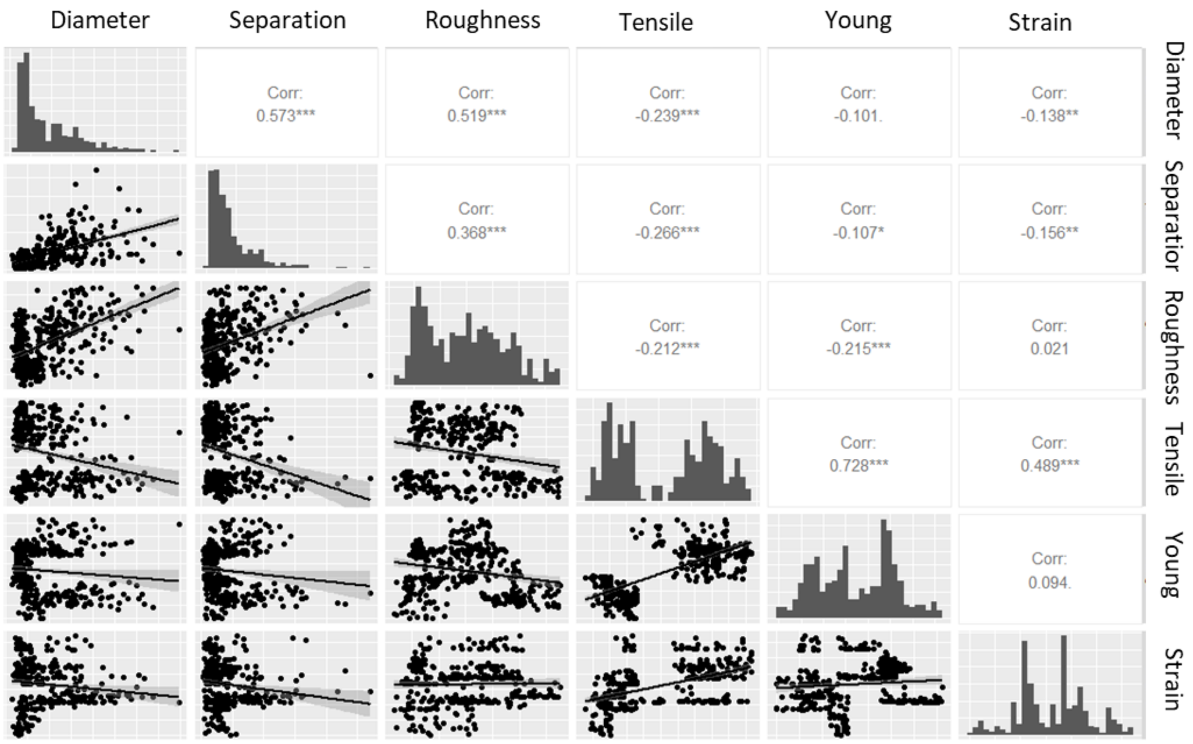

Supplementary Material Figure 2. Distribution and correlation between dependent variables

2 Decision Trees

Supplementary Material Table 3. Importance of the predictors on the output variables following DT

	Ouput variable	IMPORTANCE VARIABLE				
		HAc	dH_2O	DMSO	Diameter	Separation
**DT**	Diameter	75	12	13		
	Separation	79	8	13		
	Roughness	27	4	17	30	22
	Tensile	14	2	56	14	14
	Young	11	5	45	24	15
	Strain	12	13	39	22	14

2.1 Diameter

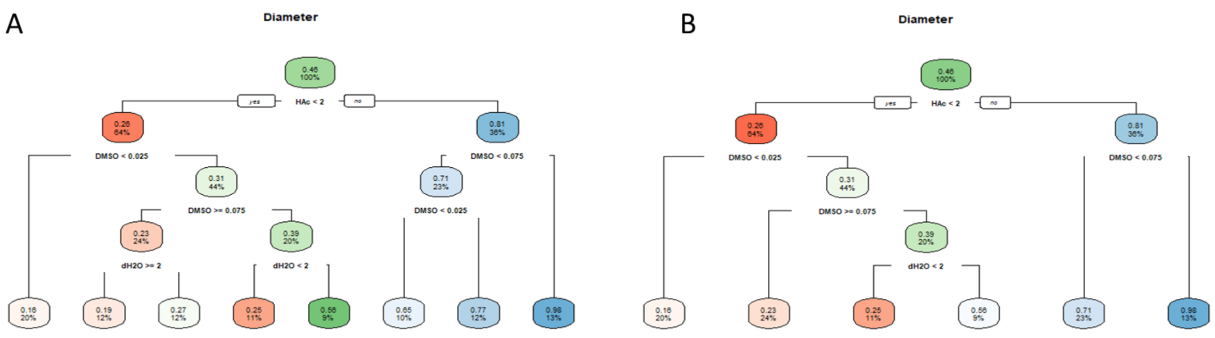

Supplementary Material Figure 3. DT Diameter. A) Complete, B) Optimased

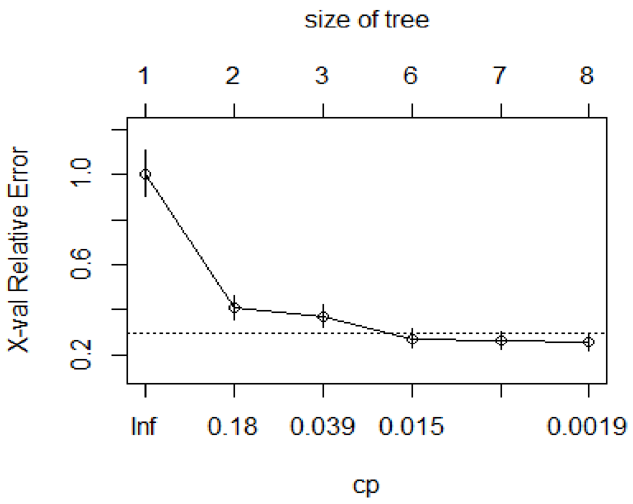

Supplementary Material Figure 4. Relative Error vs size of tree for vs complexity parameter the diameter

2.2 Inter-fibre Separation

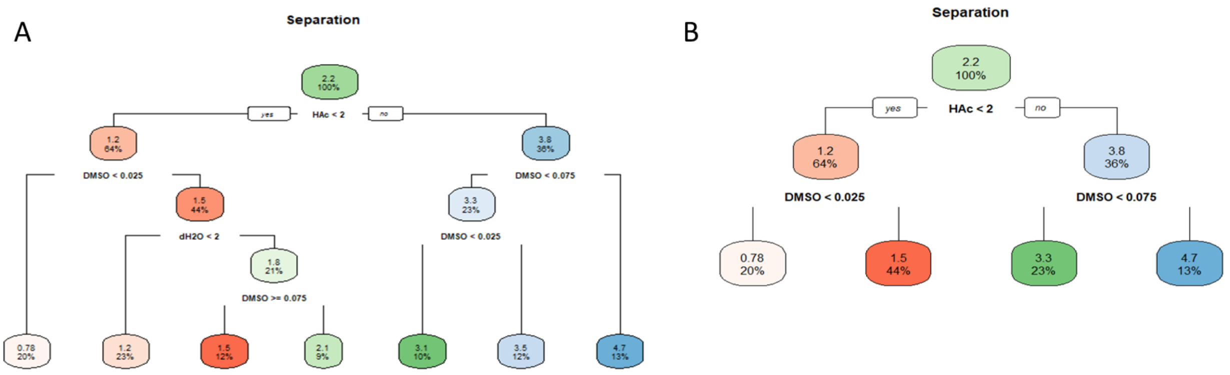

Supplementary Material Figure 5. DT Inter-fibre Separation. A) Complete, B) Optimased

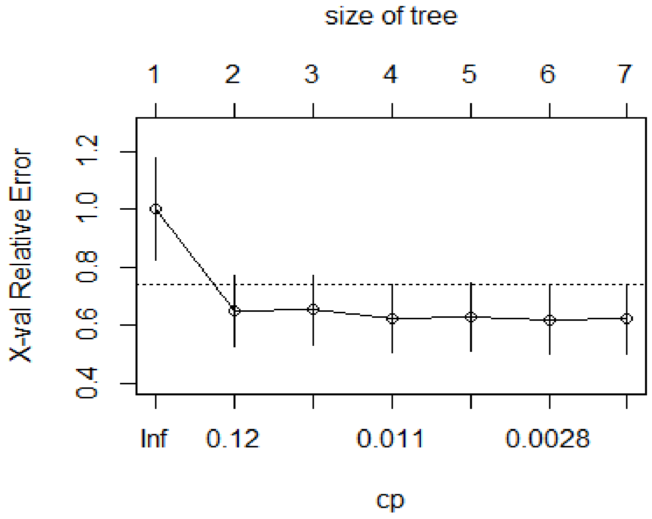

Supplementary Material Figure 6. Relative Error vs size of tree vs complexity parameter for the Inter-fibre Separation

2.3 Roughness

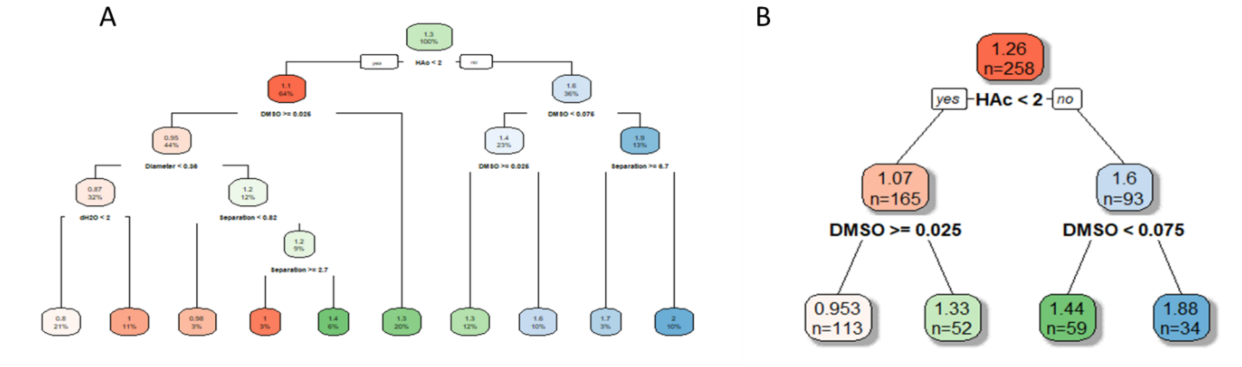

Supplementary Material Figure 7. DT Roughness. A) Complete, B) Optimased

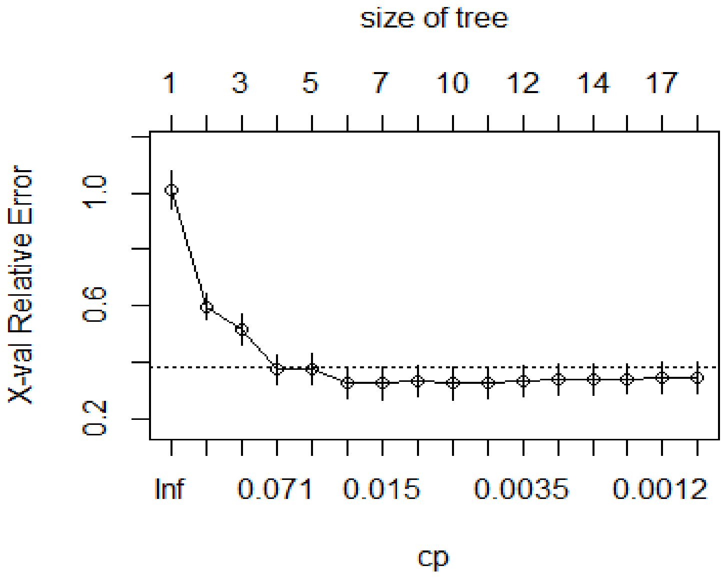

Supplementary Material Figure 8. Relative Error vs size of tree vs complexity parameter for the Roughness

2.4 Ultimate Tensile Strength

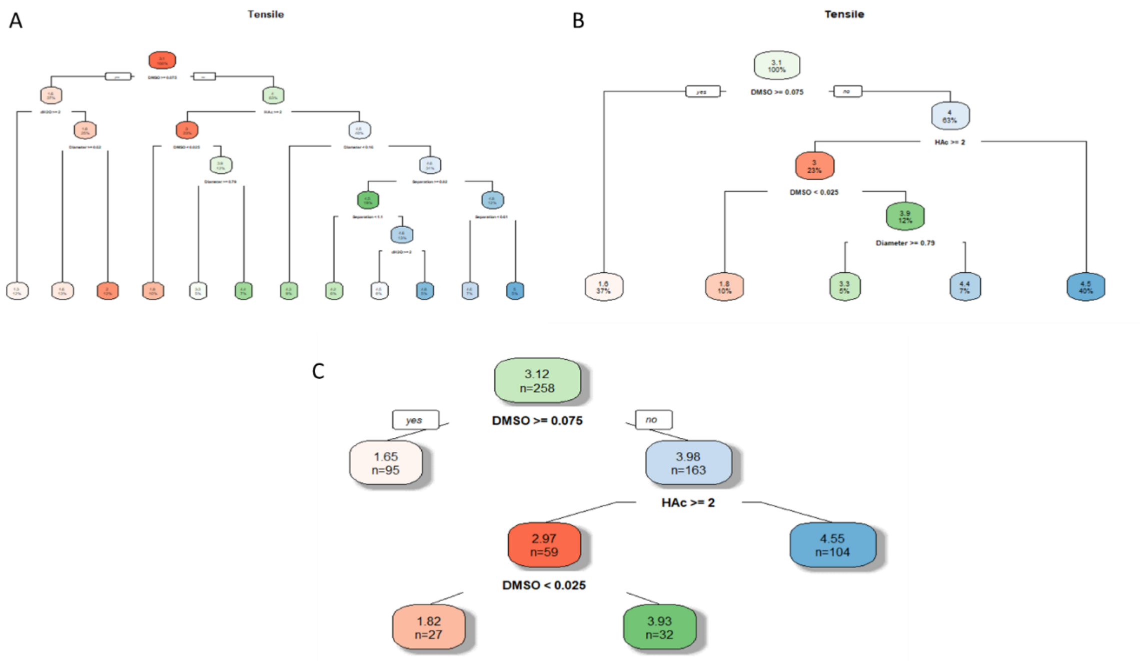

Supplementary Material Figure 9. DT Ultimate Tensile Strength. A) Complete, B) Optimased, C) Pruned

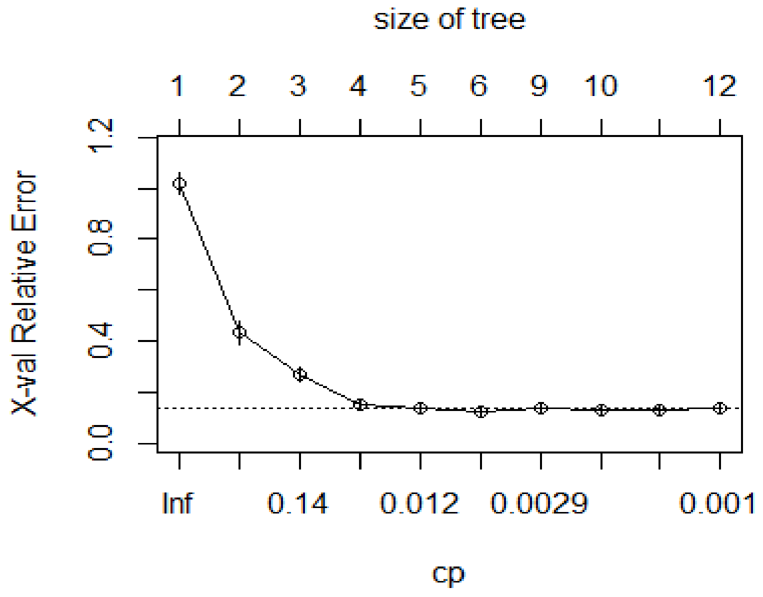

Supplementary Material Figure 10. Relative Error vs size of tree vs complexity parameter for the Ultimate Tensile Strength

2.5 Young's Modulus

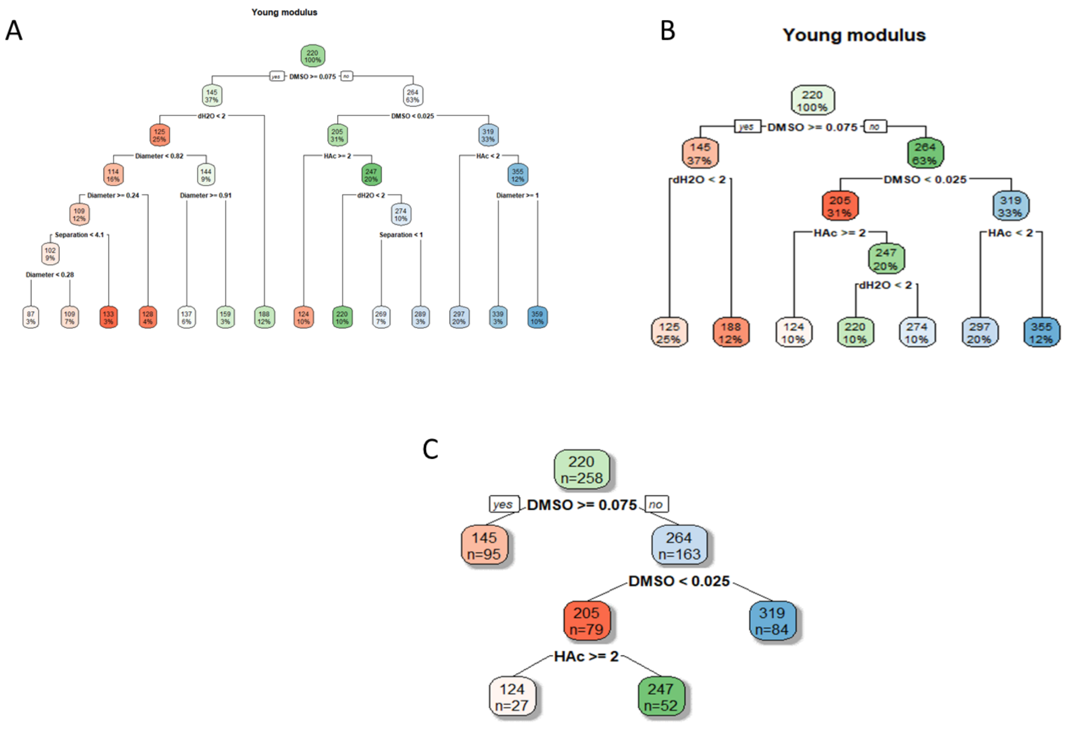

Supplementary Material Figure 11. DT Young's Modulus. A) Complete, B) Optimased, C) Pruned

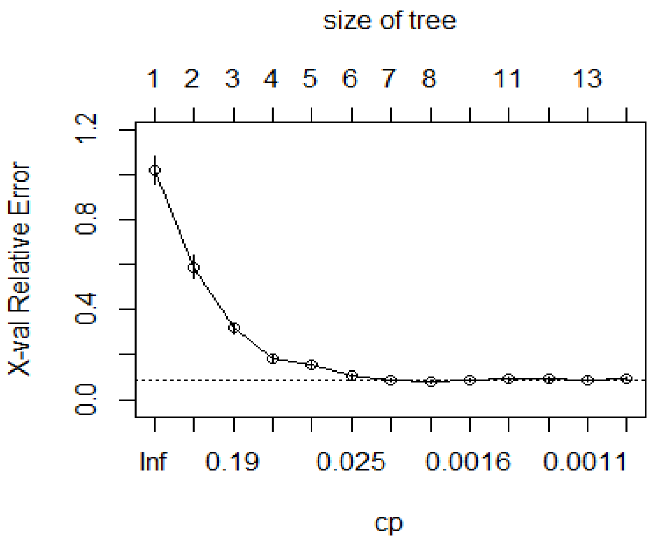

Supplementary Material Figure 12. Relative Error vs size of tree vs complexity parameter for the Young's Modulus

2.6 Strain at break

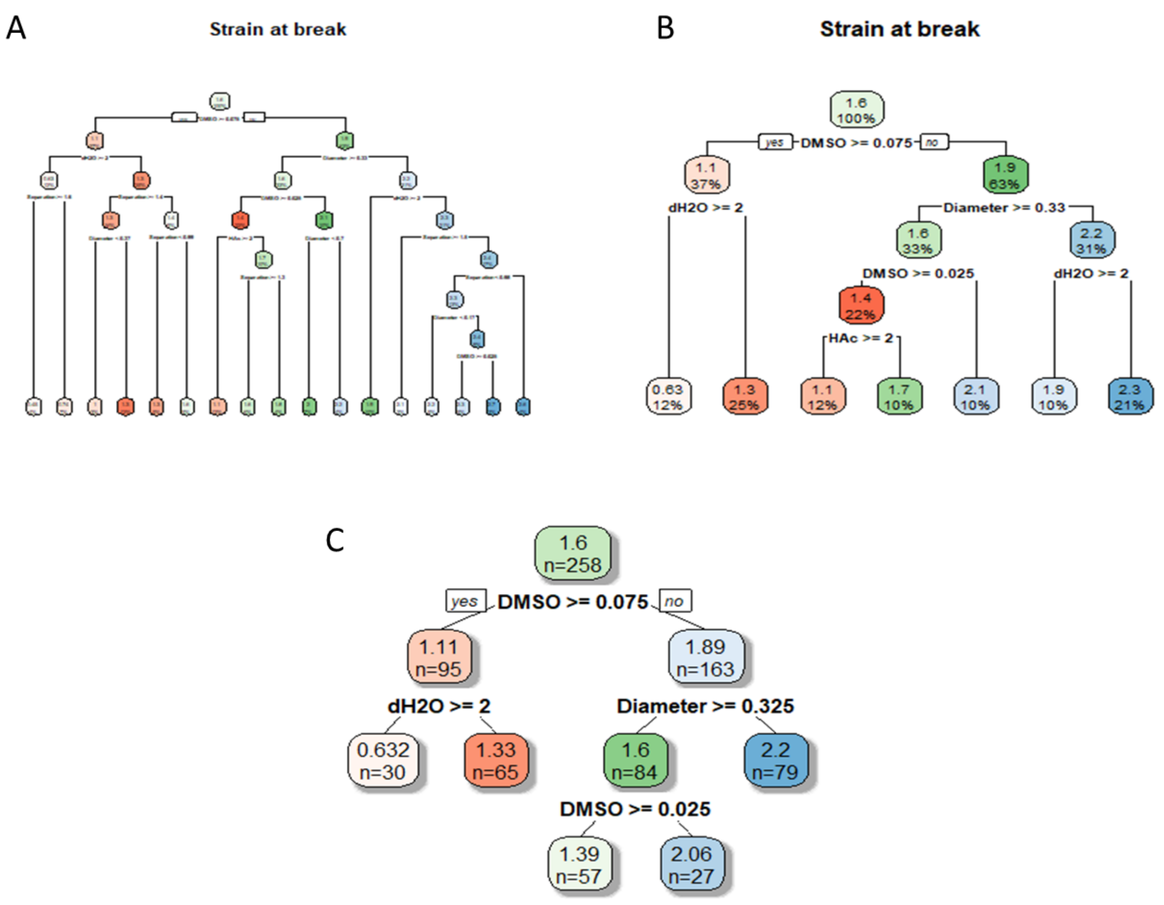

Supplementary Material Figure 13. DT Strain at break. A) Complete, B) Optimased, C) Pruned

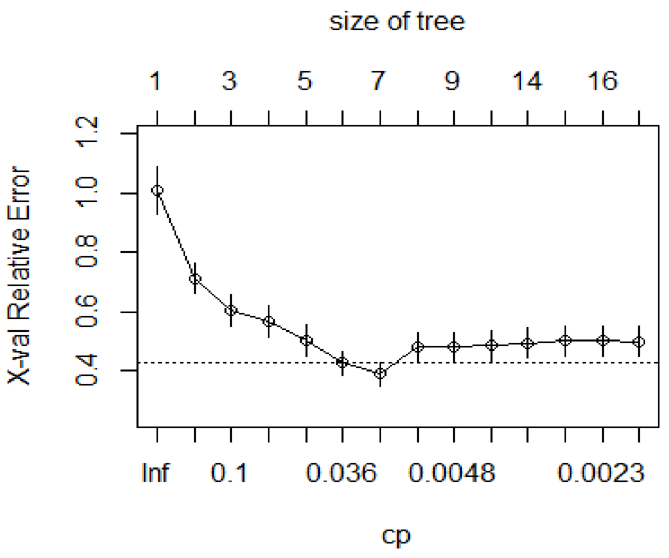

Figure 14. Relative Error vs size of tree vs complexity parameter for the Strain at break

3 Random Forest

3.1 Diameter

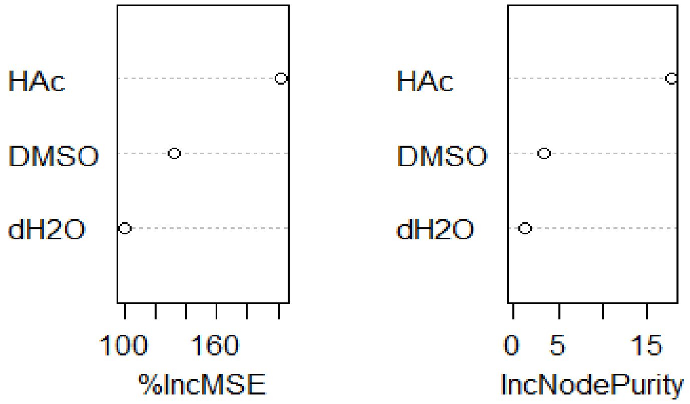

Supplementary Material Figure 15. Importance of each predictor on the Diameter

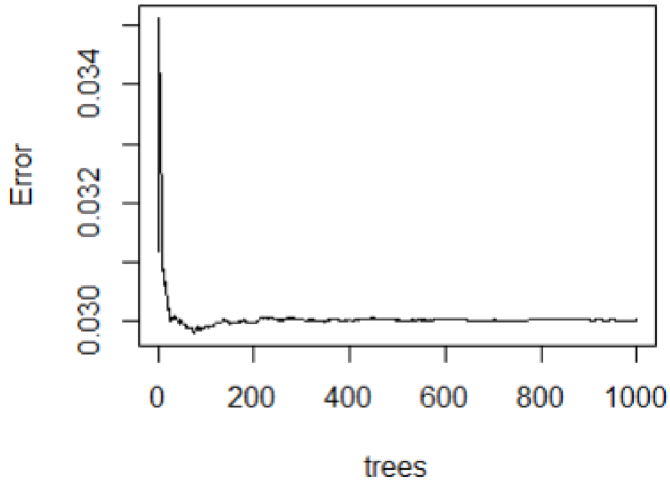

Supplementary Material Figure 16. Error vs number of trees for the Diameter

3.2 Inter-fibre Separation

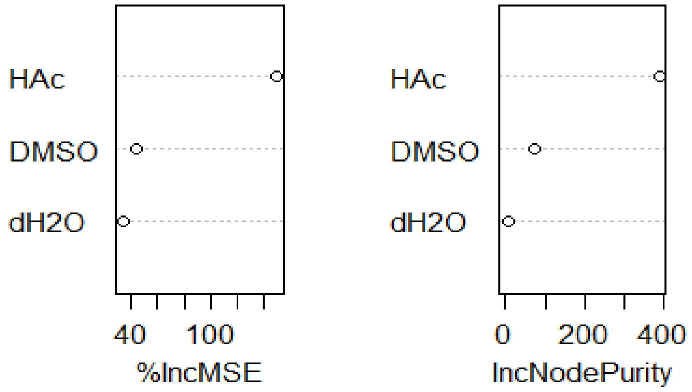

Supplementary Material Figure 17. Importance of each predictor on the Inter-fibre Separation

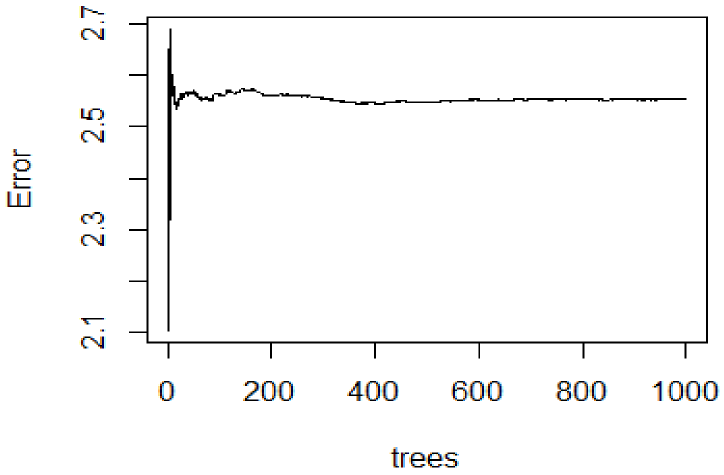

Supplementary Material Figure 18. Error vs number of trees for the Inter-fibre Separation

3.3. Roughness

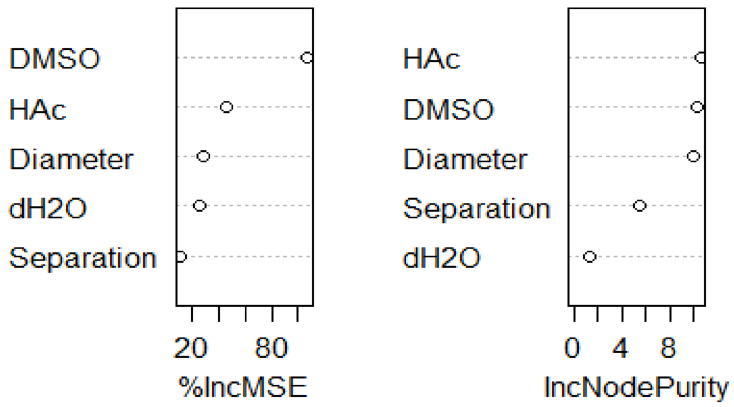

Supplementary Material Figure 19. Importance of each predictor on the Roughness

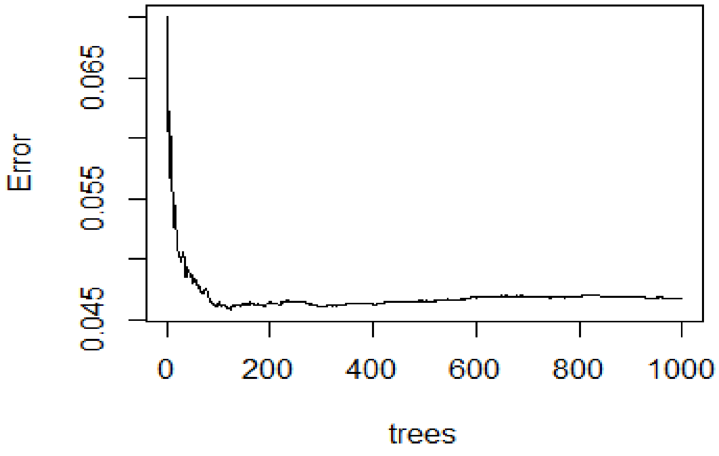

Supplementary Material Figure 20. Error vs number of trees for the Roughness

3.4. Ultimate Tensile Strength

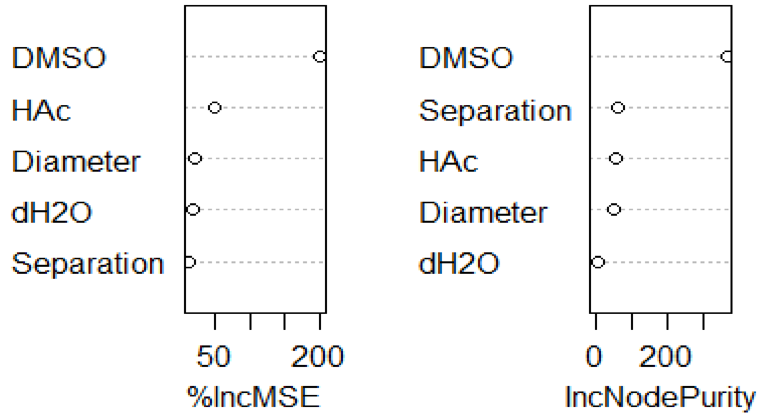

Supplementary Material Figure 21. Importance of each predictor on the Ultimate Tensile Strength

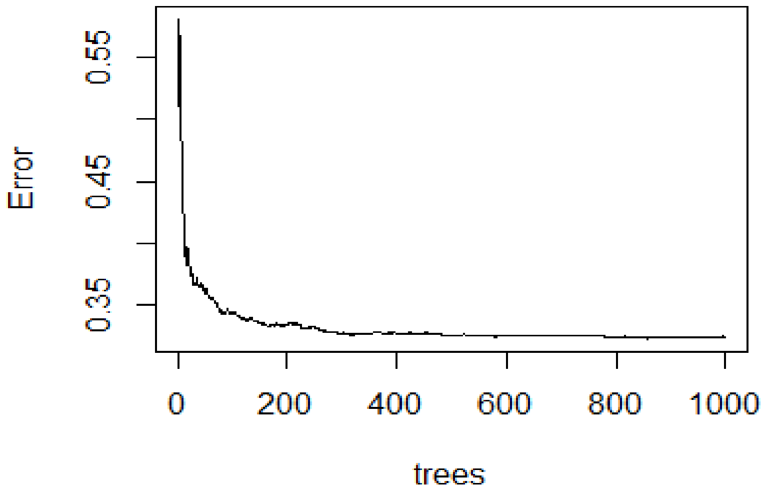

Supplementary Material Figure 22. Error vs number of trees for the Ultimate Tensile Strength

3.3 Young's Modulus

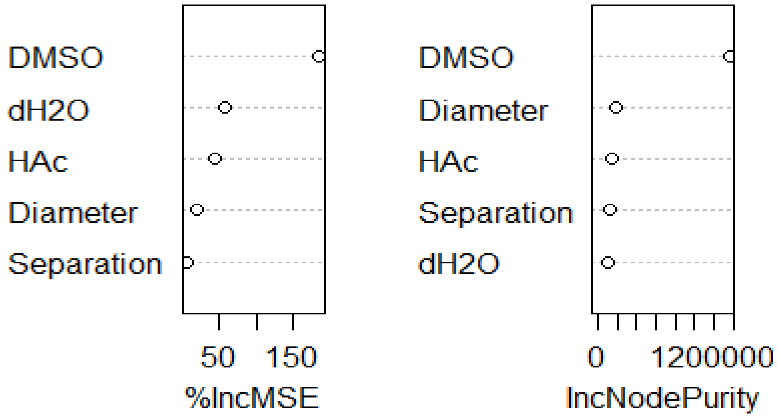

Supplementary Material Figure 23. Importance of each predictor on the Young's Modulus

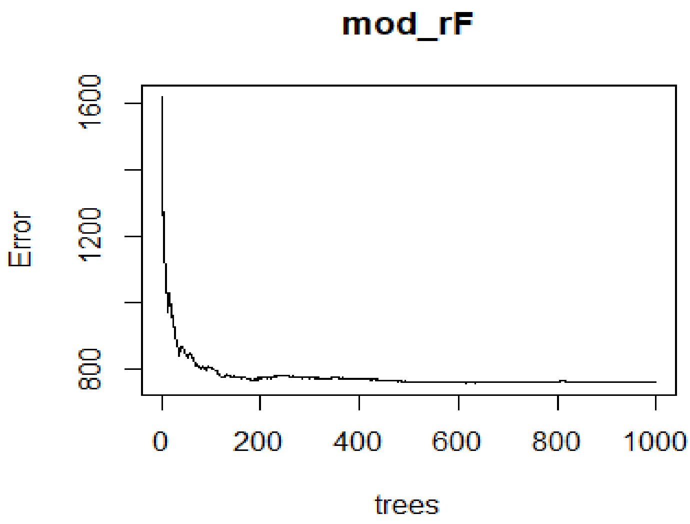

Supplementary Material Figure 24. Error vs number of trees for the Young's Modulus

3.6 Strain at break

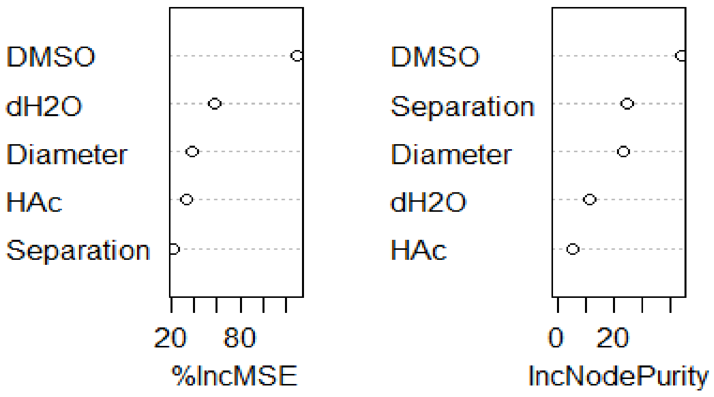

Supplementary Material Figure 25. Importance of each predictor on the Strain at break

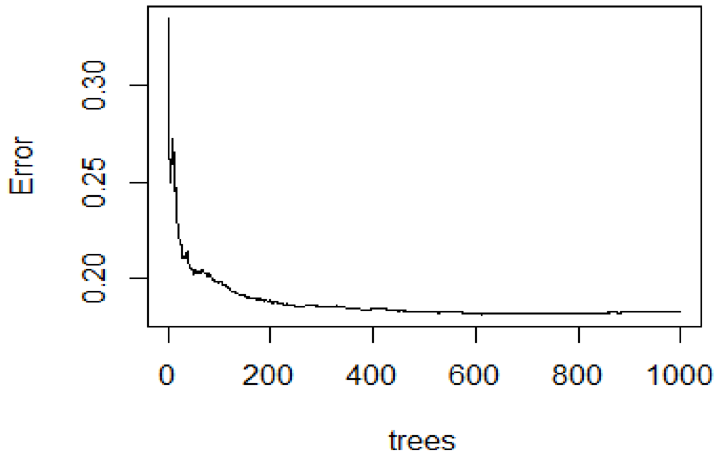

Supplementary Material Figure 26. Error vs number of trees for the Strain at break
